# Allocation strategies of savanna and forest tree seedlings in response to fire and shading: outcomes of a field experiment

**DOI:** 10.1038/srep38838

**Published:** 2016-12-21

**Authors:** Jacques Gignoux, Souleymane Konaté, Gaëlle Lahoreau, Xavier Le Roux, Guillaume Simioni

**Affiliations:** 1 Institute of Ecology and Environmental Sciences. UMR 7618 (UPMC-CNRS), 4 Place Jussieu, 75005 Paris, France; 2 Research Pole on Environment and Sustainable Development. University Nangui Abrogoua, UFR-SN. 02 BP 801 Abidjan 02, Côte d’Ivoire; 3 Ecologie Microbienne, INRA, CNRS, Université Lyon 1. UMR 5557, Université Claude Bernard Lyon I 43, Boulevard du 11 Novembre 1918, 69622 Villeurbanne cedex, France; 4 INRA, Ecologie des Forêts Mediterranéennes (UR629). Domaine Saint Paul, Site Agroparc, 84914 Avignon Cedex 9, France

## Abstract

The forest-savanna ecotone may be very sharp in fire-prone areas. Fire and competition for light play key roles in its maintenance, as forest and savanna tree seedlings are quickly excluded from the other ecosystem. We hypothesized a tradeoff between seedling traits linked to fire resistance and to competition for light to explain these exclusions. We compared growth- and survival-related traits of two savanna and two forest species in response to shading and fire in a field experiment. To interpret the results, we decomposed our broad hypothesis into elementary tradeoffs linked to three constraints, biomass allocation, plant architecture, and shade tolerance, that characterize both savanna and adjacent forest ecosystems. All seedlings reached similar biomasses, but forest seedlings grew taller. Savanna seedlings better survived fire after topkill and required ten times less biomass than forest seedlings to survive. Finally, only savanna seedlings responded to shading. Although results were consistent with the classification of our species as mostly adapted to shade tolerance, competition for light in the open, and fire tolerance, they raised new questions: how could savanna seedlings survive better with a 10-times lower biomass than forest seedlings? Is their shade intolerance sufficient to exclude them from forest understory?

Predicting the fate of ecosystems under global climate change implies predicting the movement of ecotones^1^. It is not a trivial task, since ecotones can have a fairly buffered response to climate change^2^. In the tropics, savanna areas may extend on forests or get invaded by trees, depending on many interacting factors such as, among others, atmospheric CO_2_ enrichment, afforestation, change in fire regimes or herbivore pressure^3^. The forest-savanna boundary is often very sharp^4^, the environment completely changing within a few metres, with completely different sets of species, to the point where characteristic species are used to determine precisely the boundary between the two ecosystems^5^,^6^. While water availability seems responsible for the mere existence of tropical gallery forests along temporary water streams^7^,^8^,^9^, the sharpness of the ecotone seems to be due to fire^10^, although soil differences may also play a role^11^,^12^,^13^,^14^. When intense fires are very frequent (yearly), the transition can occur within just 4 m^15^. Tree species from one ecosystem are usually only found in the other as dying or dead individuals, or as seedlings^16^. Seedlings of savanna species have a lower survival rate in forests^17^, as do seedlings of forest species in savannas^17^,^18^. Since light is the main limiting factor in forests and fire the main driver of the savanna tree community in ~50% of savannas worldwide^19^, antagonistic adaptations to these factors may explain the sharpness of the ecotone. Our aim in this study is to examine to what extent these two factors explain differences in seedling traits between savanna and forest tree species.

There are clear microclimatic differences between forests and savannas, with the forest understory having lower light availability^20^, higher air humidity, lower average temperature and daily variation in temperature^21^, lower ground temperature^12^, and lower fuel bed flamability^22^. Forest species specialisation is largely determined by light levels, from fast-growing, light demanding, and short-lived pioneer species, to slow growing, shade tolerant, and long-lived species. Savanna tree species present many adaptations to (1) frequent fires: a thicker bark^4^, a higher resprouting ability^23^, a larger root:shoot ratio and more carbohydrate reserves than forest species at the seedling stage^15^; or (2) browsing by large herbivores: spines and thorns, toxic chemicals, cage morphology^24^,^25^. Here, we will focus on fire rather than herbivores. Forest species present a faster growth, wider crowns^26^, and a bigger size at reproduction^4^. Clearly, at the adult stage, forest species should exclude savanna species through competition for light. Forest tree seedlings that reach nearby dense savanna tree clumps or thickets sometimes overgrow the savanna trees^15^,^27^. Once established, the dense shade they cast is often sufficient to outcompete grass and exclude fire, hence initiating a forest island within the savanna, that may persist for a long time and eventually merge with the nearby forest edge or other forest islands^28^,^29^. Although it does exist, this phenomenon is relatively rare. Apparently, forest species are excluded from the savanna by fire at a very early stage^18^, even before their competitive growth advantage shows up.

Gignoux *et al*.^30^ proposed that there could be a tradeoff between ‘competitive ability’ and ‘fire resistance’ among savanna young trees older and larger than seedlings, based on the evidence that surviving fire and competing for light required opposite biomass allocation patterns: to resist fire, a young tree has to invest a lot of biomass in its belowground system in order to resprout quickly after fire, while to survive competition for light in an open environment, a young tree has to invest a lot into a tall stem which will overtop its neighbours (and grass). Further, for the same biomass investment, a more conical main stem reduced the loss of biomass through topkill by fire at the cost of a slower growth in height, while a more cylindrical stem sped up growth in height but increased the amount of biomass lost through topkill^30^. Seedlings are even more constrained, as they must build up root reserves sufficient to survive by resprouting after their first fire in, at the extreme, just one growing season^18^. This constraint may explain why forest tree seedlings are unable to survive in savannas beyond their first fire.

Tree species from one ecosystem can also present different strategies. For example, forest pionneer species are usually quite good at colonizing open areas, before being replaced by other species able to grow in the shade. In savannas, trees from some species can grow isolated, while those of other species are never found alone. Clearly, competing for light in an open environment (savanna or early forest succession) requires different adaptations from surviving to dense shade under a closed canopy. In forests, we may expect trees either adapted to dense shade or to competition for light in an open environment; in savannas, we may expect trees either adapted to competition for light, or to frequent fires.

Altogether, these environmental constraints actually yield more than a single tradeoff: we identified six plant features for which competition for light, shade tolerance and fire resistance imply contrasted, optimal traits (Table 1). For example, a tree seedling cannot be both prostrate and erect, or have a low photosynthetic compensation point and a high maximal assimilation rate. When competition for light in the open is the main environmental constraint, we expect tree seedlings to optimize their growth in height in order to quickly overtop their neighbours and avoid being overtopped themselves. As a result, we expect them to allocate more biomass into stems and leaves, to have a cylindrical stem, a high maximal assimilation rate, and an erect stem with little ramification. When growing under dense shade, the priority should be to balance the carbon budget at any cost, by having a low photosynthesis compensation point, and to maximize exposition to light by investing into leaves and display them avoiding self-shading. Finally, frequent fires mean frequent topkill, specially at the seedling stage, and we expect this to produce trees investing into belowground storage organs, having a conical stem, possibly erect, to avoid fire damage, and with a high level of ramification to improve light capture.

We built a manipulative field experiment on two savanna and two forest tree species, with two controlled factors, fire and shading, in order to (1) compare the performance of the savanna and forest species under the shading and fire constraints and relate it to their trait values, and (2) test whether they behaved as expected from Table 1 given the environmental constraint they usually face.

## Material and Methods

### Study site and study species

The experiment was conducted at the Lamto tropical ecology research station, in Côte d’Ivoire (6°13′N, 5°02′W). The typical vegetation is a mosaic consisting of a matrix of Guinea savanna (wet savanna characterized by Andropogoneae grasses, short trees and taller *Borassus aethiopum* palm trees) interspersed with gallery forests (rainforest tall trees) along permanent and temporary water streams. The climate is four-seasonal, including a long dry season (November-February), a long rainy season (March-July), a short dry season (one month over the AugustSeptember period) and a short rainy season (September-October) with an average yearly rainfall of 1150 mm (Appendix 1). Fire occurs yearly in the middle of the long dry season (January). The soils are tropical ferrugineous soils with very low nutrient availability. There are very few herbivores in this environment (~500 kg/km^2^). For more details, see Menaut & César^8^ or Abbadie *et al*.^13^.

Because of the numerous tradeoffs involved in Table 1, we focused on a few common species found in savannas or forests, in a controlled experiment with in-depth measurements. We selected two savanna species and two forest species, based on their abundance, the possibility to grow them from seeds, and when possible their observed contrasted behaviors, to have better chances to highlight what traits are involved in the ability to survive in one ecosystem or the other. The savanna species belonged to the group of four dominant species representing 90% of the savanna tree biomass^8^, and hence can be considered to represent roughly 45% of the tree layer: *Bridelia ferruginea* (Afzel. ex G. Don) Benth. (Phyllanthaceae) and *Piliostigma thonningii* (Schumach.) Milne-Redhead (Caesalpiniaceae). They can reach heights of 10–12 m^8^. The two forest species have different ecologies: *Ceiba pentandra* (L.) Gaertn. (Malvaceae) is a 40–60 m tall species, planted or spontaneously growing in rainforests and gallery forests^31^, and frequently encountered as seedlings in the savanna^32^; *Cynometra megalophylla* Harms (Caesalpiniaceae) is one of the most common, shade-tolerant, gallery forest species^15^,^32^, reaching heights of 15–22 m^33^. In Lamto, *Ceiba* behaves as a pioneer or ‘early successional’ species that can later dominate gallery forests thanks to its size; its status in other parts of the world is unclear. *Cynometra megalophylla* is evergreen, while the other species are deciduous.

### Experimental design

The experiment consisted in controlling the light level and occurrence of fire on two 12 × 18 m plots of shrubby savanna where seedlings were grown. The light level was controlled by having a double layer shade cloth (green mosquito net) set at 2 m height above one of the plots, the other being left in full light. This shade level corresponds to 40% full light, a level commonly encountered in tree clumps in the savanna^13^,^34^. In order to focus on tree species traits in a standard environment, we reduced competition by removing the grass from the experimental plots prior to plantation. Half of each plot was subject to fire after the first growing season. Dry grass fuel amounting to the usual average fuel load in Lamto (1000 g m^−2^)^13^ was spread on the half plots for burning. A metal sheet was set in the ground to prevent the fire from reaching the unburnt other half plot.

432 seedlings grown for three months in nursery were transplanted on the experimental plots in June, during the long rainy season. In each fire × light treatment sub-plot, we installed 27 seedlings of each study species in three randomized 3 × 3 m blocks, allowing 1 m between seedlings. Seedlings dying during the first two weeks after transplantation were replaced. Plots were regularly weeded by hand. Fire occurred at age 10 months. Shade cloths modified the microclimate of the plots, so that the shaded plot had a higher topsoil gravimetric water content at the end of the long rainy season (12.3% vs 9.6% in September).

### Selection of relevant traits

**Figure 1:**
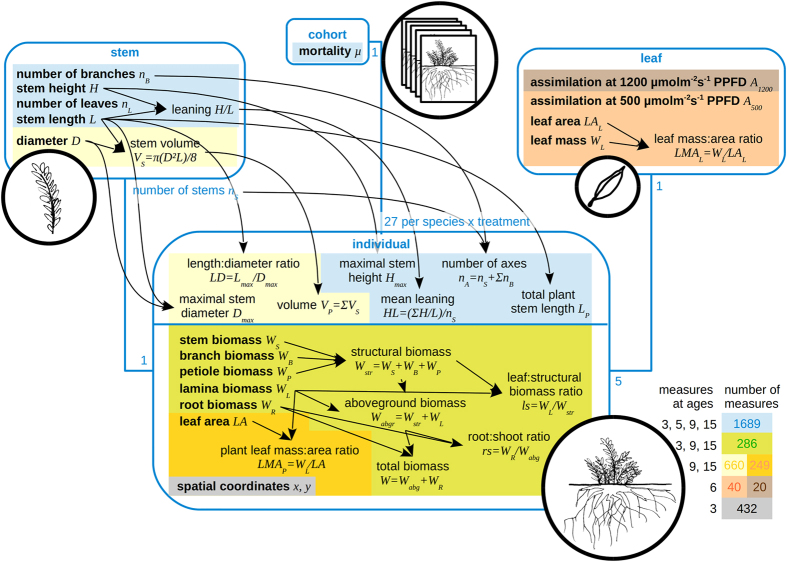
Graphical description of the plant measurements. Blue boxes are the experimental units (stems, individuals, cohorts), and blue lines indicate the relations between them with multiplicities (e.g. an individual has *n*_*S*_ stems, a cohort has 27 individuals, one leaf was sampled on five individuals). Black text are the variables. Black arrows show dependencies among variables (e.g., maximal stem diameter was computed from all stem diameters for each individual); measured variables are in bold whereas computed variables are in standard font. Background colors show at which dates measurements were made, with the colored numbers in the legend entries showing how many measures were available for each variable set. All variables below the blue line in the ‘Individual’ box and individual leaf area and biomass for sampled leaves were destructive (individuals were sampled). All other variables were repeatedly measured/computed on the same individuals over time.

To evaluate the performance of seedlings under the experimental treatments, we measured (1) survival probability; and (2) growth, through various non-destructive (stem length, diameter…: cf. Fig. 1 for the full list) and destructive (stem, leaf, root biomasses: Fig. 1) size variables. Because biomass is a destructive measurement, we had to use non-destructive size variables to estimate through multiple linear regression *relative growth rate (RGR*), a common measure of plant intrinsic growth ability, as *RGR* requires a time series of biomass values for the same individual (Appendix 2). *RGR* was computed as *RGR* = *d(Ln(W*))/*dt* ≈ (*Ln(Ŵ*_*t*+*Δt*_) − *Ln(Ŵ*_*t*_))/*Δt*, where *Δt* represents the time between two measurement dates (at ages 3, 9 and 15) and *Ŵ* is the total *estimated* plant biomass. Using average seed weights (Appendix 3), we were able to also compute initial *RGR*, between ages 0 and 3.

To test if our study species behaved as expected given their ecosystem of origin (Table 1), we measured the relevant traits listed in Table 1 (Fig. 1). We considered the *root:shoot ratio (rs*) and the *leaf:structural biomass ratio (ls*) as the most relevant biomass ratios (Table 1). Plants have been reported to have a higher *rs* when water- or nutrient-stressed^35^,^36^ and a lower one when competing for light^37^. *ls* measures the investment into the productive photosynthetic system relative to its mechanical support^38^. Depending on their size, seedlings in tropical forests have been reported to either increase or decrease their leaf biomass in shade compared to high light levels^39^. We computed *biomass* a*llocation coefficients* 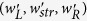 as: 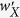 = *dW*_*X*_/*dW* ≈ (*Ŵ*_*X,t*+*Δt*_ − *Ŵ*_*X,t*_)/(*Ŵ*_*t*+*Δt*_ − *Ŵ*_*t*_). First, biomass of leaves, roots and structural biomass were estimated from non-destructive variables (Appendix 2). Then, allocation coefficients at the species × treatment level were computed as the slopes of regressions of (*Ŵ*_*X,t*+*Δt*_ − *Ŵ*_*X,t*_)s on (*Ŵ*_*t*+*Δt*_ − *Ŵ*_*t*_). The *stem length:diameter ratio (LD*) measures stem taper with length and is linked to fire resistance^30^. Maximal assimilation rate *A*_*max*_ at high and low light irradiance is a convenient way to test the saturating region and the increasing region of the photosynthesis response curve to light. The *stem height:length ratio (HL*) is a measure of tree leaning, which is important with regard to fire damage to trees (leaning trees are more frequently debarked on the down facing side of their trunk^40^). *Leaf mass:area ratio* for the whole seedling (*LMA*_*P*_) is negatively correlated to relative growth rate and shade tolerance^41^.

### Raw measurements on seedlings

Measurements on seedlings comprised non-destructive and destructive variables. Non-destructive variables were measured at the leaf, stem, whole plant, and cohort level (see Fig. 1 for the whole list of variables and their dates of measurements). Height and stem lengths were measured to the nearest 0.5 cm and stem diameters to the nearest 0.1 mm with a calliper. Leaf assimilation (μmol m^−2^ s^−1^) was measured on the top, last fully developed leaf of 5 individuals of each species × light treatment using a LICOR-6400 gas exchange analyser (LI-COR, Inc., Lincoln, NE USA). Leaf assimilation was measured at both high irradiance (1200 μmol m^−2^ s^−1^) and low irradiance (500 μmol m^−2^ s^−1^) for leaves grown in the full light treatment, and at low irradiance only (500 μmol m^−2^ s^−1^) for leaves grown in the shaded treatment. The irradiance values were chosen as representative of (i) the linearly increasing and (ii) the saturation regions of the assimilation reponse curve according to previous data obtained for other tree species at Lamto^42^.

Destructive variables were measured at the whole plant level. Three seedlings per 9-seedling block were randomly selected for destructive sampling (at age 3, seedlings were actually selected directly from the nursery). They were carefully uprooted. Roots, leaf blades, petioles, main stem, and branches were separated, oven-dried for 48 hours and weighted to the nearest 0.1 mg. At age 9, all leaves of each sampled seedling were photocopied. Total leaf surface was then calculated as the product of the total weight of photocopies and the surface/weight ratio of the paper (80 g m^−2^ Fig. 1). At age 15, the same method was used but on a maximum of 25 leaves per plant (as some had hundreds of leaves), and the total number of leaves was obtained by multiplying the average leaf surface by the total number of leaves.

### Meta-analysis of allocation ratios

As our experimental approach only concerned four species, we used published data^43^,^44^,^45^,^46^ on tropical tree seedling biomass measurements and original data kindly provided by W. Hoffmann^4^ to give our results a broader context. All these studies worked on tree seedlings of an age between 0 and 200 days, subject to various treatments (light level, nutrients, fire, drought…). We computed allocation coefficients to root, leaf and structural biomass for every species x treatment combination, as our aim was only to display a large-scale pattern of real values. The studies all used seedlings grown from seeds or transplanted, except one that used seedlings naturally grown in their environment. The data represent 23 forest and 62 savanna species from tropical parts of Africa, South America and Asia.

### Statistical analyses

All analyses were performed using the R^47^ software. Nineteen variables were measured at the individual plant level, but on different numbers of plants (Fig. 1): *W, W*_*abg*_, *W*_*R*_, *W*_*str*_, *W*_*L*_, *LA, L*_*P*_, *n*_*L*_, *H*_*max*_, *n*_*A*_, *D*_*max*_, *V*_*P*_, *HL, LD, LMA*_*P*_, *rs, ls, A*_*500*_, *A*_*1200*_. One variable was estimated at the individual plant level: *RGR.* Four variables were estimated at the species × treatment level: *w*′_*L*_, *w*′_*str*_, *w*′_*R*_, *μ*. The main set of variables (all the individual-level variables except *A*_*500*_ and *A*_*1200*_) was analysed in the same way. We first assessed the correlations among them through a principal component analysis on the centered reduced data (cor() and prcomp()R functions). Because of missing data and of the mix of destructive and non-destructive measurements (Fig. 1), there were only 254 observations available for this analysis. To take advantage of the whole dataset as much as possible, we then performed separate analyses of variance for each variable.

We considered the following experimental treatments: ecosystem type (forest vs. savanna), species within ecosystem, shading, and burning. The experimental design changed from date to date: the first seedlings were sampled before planting, as a reference point before treatment application. Hence at age 3, ecosystem and species were the only relevant factors for analysis. At ages 5 and 9, the fire treatment had not yet occurred, so the relevant factors were ecosystem, species and shading treatment. At age 15, we should have analysed all three factors in a full factorial design, but because of deaths due to fire and other technical problems, the design became strongly unbalanced, with some treatment combinations missing. This design did not fit into standard analysis of variance modelling.

We proceeded to the analysis by (1) combining all factors into a ‘flat’ factor with 36 levels and (2) using contrasts to address more specific questions of interest (see Appendix 4 for details on the statistical modelling). The analysis was further complicated by the existence of repeated measurements on individual plants for the non-destructive variables (*L*_*P*_, *n*_*L*_, *H*_*max*,_ *n*_*A*_, *D*_*max*_, *V*_*P*_, *HL, LD, RGR*) but not for the destructive variables (*W, W*_*abg*_, *W*_*R*_, *W*_*str*_, *W*_*L*_, *LA, LMA*_*P*_, *rs, ls*). We used mixed linear models (R functions rls() and lme()) to account for that when required (appendix 4). Finally, we accounted for possible spatial variation due to plot heterogeneity by recording the location of every seedling and fitting a quadratic response surface within every statistical model.

Allocation coefficients 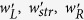 were estimated at date 9 through covariance analysis lm() function with species and shading as factors), with stepwise simplification to the simplest models.

The survival of seedlings at the end of the experiment was analysed through a stepwise logistic regression (R glm model with binomial error and logit link function).

## Results

### Preliminary: effects of space and time and correlation structure

**Figure 2:**
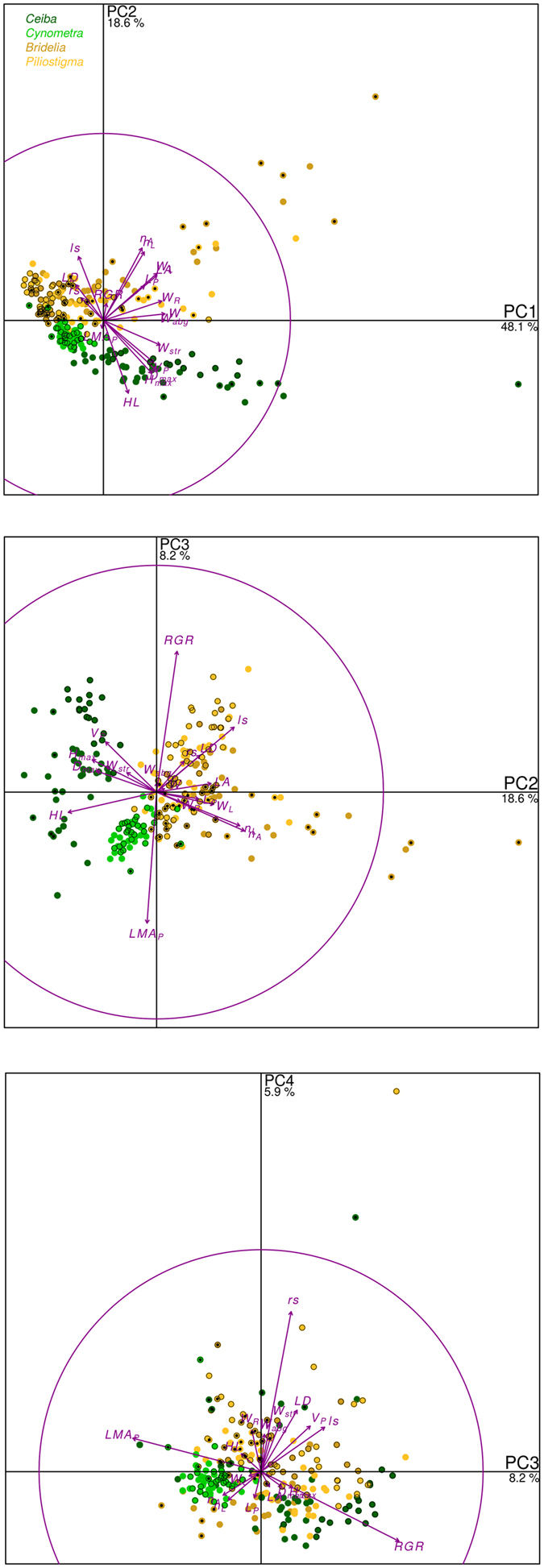
Principal component analysis of the 18 main individual-plant level variables (cf Fig. 1 for the full list). The four first principal components accounted for 80.8% of the total variance (value for each axis shown under the axis label). Symbols: location of the 254 individual observations; arrows: locations of the original variables in the correlation circle; symbols with a black border: individual grown under shade; symbols with a central black dot: burnt individuals.

There were strong correlations (Table 2) among all biomass variables and most non-destructive size variables; trait ratios (*HL, LD, rs, ls*) were less strongly correlated among themselves and with the size variables; *LMA*_*P*_ and *RGR* were almost uncorrelated to any other variable. These patterns were obvious on the PCA results (Fig. 2):

- the first component axis (48% of the total variance) was positively associated with *W, W*_*abg*_ and *W*_*R*_, slightly to *W*_*str*_, i.e. it is clearly a whole plant size axis;
- the second axis (19% of variance) opposed *LA, W*_*L*_, *n*_*A*_ and *n*_*L*_ on its positive end to *HL, D*_*max*_ and *H*_*max*_ on its negative end. It is associated to the relative investment into leaves vs. stems. Three groups of points were separated by this axis: *Ceiba* individuals on its negative end, then *Cynometra*, and then a mixed group of the two savanna species on its positive end;
- the third axis (8% of variance) opposed *RGR* to *LMA*_*P*_;
- the fourth axis (6% of variance) was strongly positively associated to *rs*.

Whereas species groups were obvious in the 1–2 and 2–3 planes, the experimental factors did not group consistently in the PCA space (Fig. 2).

The spatial terms introduced in all analyses of the individual variables (Table 3) were sometimes significant but never explained more than ~5% of total variance (Appendix 4, Fig. A4.1).

For all size variables except *L*_*P*_, there was a significant positive effect of age (Table 3). Biomass variables tended to increase by 2 orders of magnitude (100-fold), while most linear dimensions (e.g. stem length or diameter) increased 10-fold (Appendix 4).

### Performance of seedlings under experimental treatments

We assessed seedling performance by looking at the dynamics of total biomass *W* (since all other size variables were strongly correlated to *W*), of the relative growth rate *RGR*, and of the survival rate (1-*μ*).

**Figure 3:**
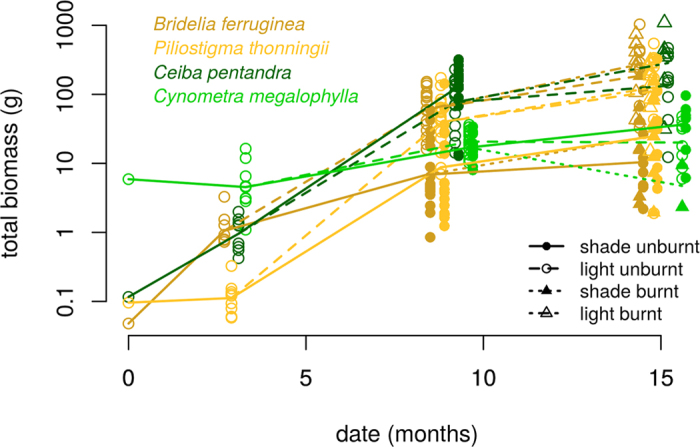
Dynamics of total seedling biomass in response to treatments. At age 0, we plotted the seed biomass (Appendix 3). Fire occured at age 10 months. At ages 3, 9 and 15 months, x-axis values were spread to improve readability.

For *W*, there was no significant difference between ecosystems (Table 3 and Fig. 3). Savanna species strongly responded to shading, but not forest species. There were significant differences between *Bridelia* and *Piliostigma* in full light, and between *Ceiba* and *Cynometra* whatever the light treatment. Biomass did not respond to fire. Overall, *Cynometra* individuals reached biomasses smaller by a factor ~5 than the other three species, who were able to reach similar sizes, depending on treatment (Fig. 3).

There was a significant effect of ecosystem on *RGR* (Table 3). Using seed mass to compute the initial *RGR* (Appendix 3), *Cynometra* was the slowest grower (Fig. 3 and A4.1), not even making up for its initial seed mass after 3 months of growth. Only forest species presented a significant (negative) response of *RGR* to fire (Fig. A4.1).

**Figure 4:**
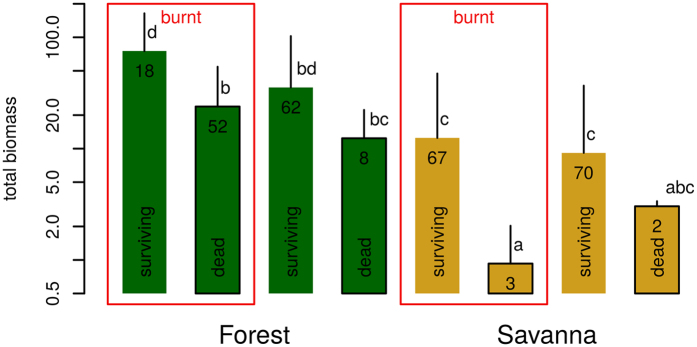
Difference in total *estimated* biomass between dead and alive trees of the savanna and forest species groups. Biomass data was estimated from non-destructive measurements at age 9 months and survival observed at age 15. Error bars represent ± 1 standard deviation. Numbers on bars are the numbers of individuals in each group. Bars with the same letters are not signficantly different at the 5% level (groupings based on the Tukey honest significant differences method), from a linear covariance analysis model predicting log(*W*) from ecosystem*fire*status, and posterior Tukey HSD tests. Individuals not in red boxes were protected from fire.

All burnt individuals were topkilled. There was almost no mortality during the wet season until age 9 (Appendix 5, Fig. A5.1). The dry season mortality (unburnt plots between ages 9 and 15) was significant and similar for all species (36%), and did not depend on the shade treatment. There was an additional mortality due to fire for forest species only (burnt plots between ages 9 and 15): mortality reached 69% for *Ceiba* and 95% for *Cynometra*. The simplest model predicting survival probability from other variables and experimental treatments included ecosystem, fire and total biomass *Ŵ* (Appendix 5, Table A5.1). For both ecosystems, larger seedlings had a higher probability of survival to fire (Fig. 4), while there was no significant effect for unburnt seedlings. The fraction of biomass allocated to roots did not have a significant effect on survival. Savanna seedlings experienced better or equivalent survival rates as forest seedlings with a biomass smaller by an order of magnitude.

### Trait differences between ecosystems and species

**Figure 5:**
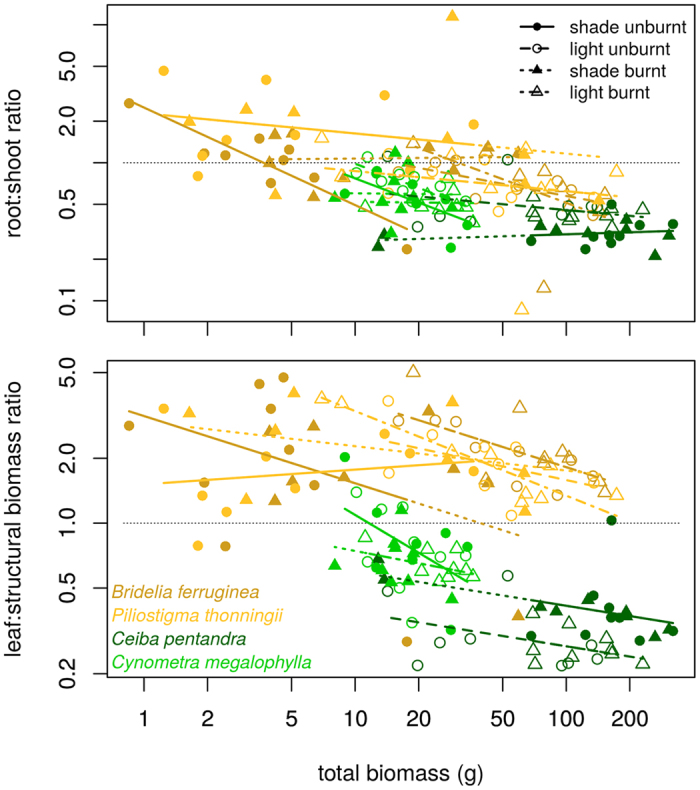
Root:shoot ratios (top) and leaf:structural biomass ratio (bottom) as functions of seedling biomass. Structural biomass includes aerial parts other than leaves. Lines show best fit regressions from a covariance analysis model (overall significant, although some individual slopes and intercepts are not). One outlier was removed from the bottom graph, corresponding to a completely defoliated seedling.

**Figure 6:**
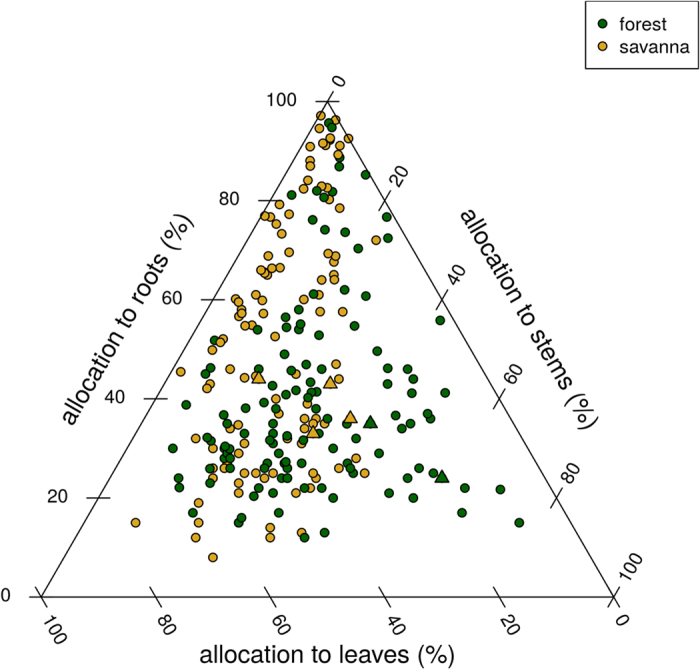
Comparison of allocation coefficients of savanna and forest tree species. Triangles: this study (at age 9); circles: data extracted from refs [43, 44, 45 and 46], and data from [4] kindly provided by W. Hoffmann.

The root:shoot ratio (*rs*) decreased with increasing biomass across species. It could be above 1 only for savanna species (Fig. 5): *Piliostigma* always had the highest *rs*, followed by *Bridelia, Cynometra* and then *Ceiba*. There was little or no response of *rs* to fire. There were clear differences between savanna and forest species regarding the leaf:structural biomass ratio *ls* (Fig. 5), which was above 1 for savanna species, and below 1 for forest species. Fire only increased *ls* for *Piliostigma* grown under shade. *Ceiba* reached the highest values for *W*_*str*_, *V*_*P*_ in shade, and *H*_*max*_ without fire, followed by *Cynometra*, and by the two savanna species (Fig. A4.1). It also had the highest values of *LA* and *n*_*L*_ in shade, while savanna species, and specially *Bridelia*, had the greatest values in full light. Finally, *Ceiba* always had the longest stem *L*_*P*_, all other species being roughly equal, except at date 3 where *Cynometra* had the longest stems, and in the burnt and full light treatment where savanna species produced the longest stems. Allocation coefficients (Table 4) confirmed and summarize these patterns: savanna species invested on average 32% of their new growth into leaves, 27% into stems and 40% into roots; while forest species invested respectively 18%, 51% and 32%. Savanna seedlings grown in full light tended to invest more growth into leaves than those grown in the shade, while forest seedlings showed little or no response to the shading treatment. Allocation patterns of seedlings of tropical trees of 85 species grown under various experimental conditions largely overlapped (Fig. 6). The only clear pattern in this figure is that only forest species may allocate more than 40% of their new biomass to stems.

The length:diameter ratio (*LD*) of the main stem varied between ecosystems and species within ecosystems (Table 3). It was smallest for *Ceiba*, and similar for the other species, except in shade where savanna species reached higher values than those of *Cynometra* (Fig. A4.1). *Ceiba* always had the largest *D*_*max*_, followed by *Cynometra* and the two savanna species in the shade; in full light, savanna species had higher or similar values to *Cynometra*’s.

**Figure 7:**
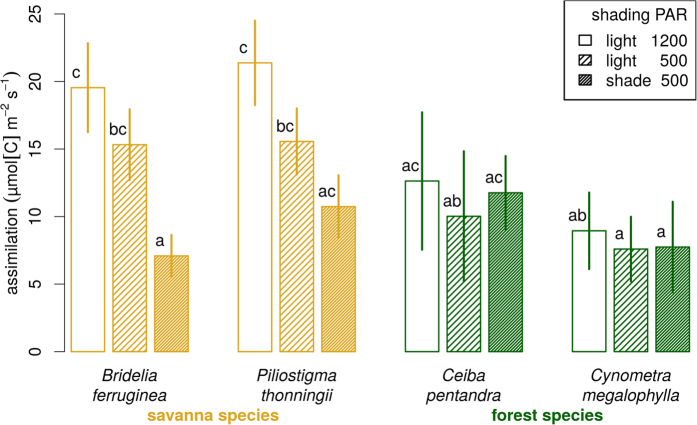
Net assimilation rate at age 6 months of seedlings grown in the open plot (‘light’) or in the shaded plot (‘shade’). Assimilation measurements were made at 1200 and 500 μmol m^−2^ s^−1^ of photosynthetically active radiation (PAR) (respective variables *A*_*1200*_ and *A*_*500*_). *A*_*1200*_ was not measured on seedlings grown in shade as it was assumed meaningless to expose shade leaves to full light. For *A*_*1200*_, an ANOVA model with only species effect was fitted with all effects found significant at the 5% level, with R^2^ = 0.72. For *A*_*500*_, an ANOVA model with species and shade effects and their interactions was fitted, and all effects were found significant at the 5% level, with R^2^ = 0.60. Error bars represent ± 1 standard deviation (n = 5). Bars with the same letters are not signficantly different at the 5% level (groupings based on the Tukey honest significant differences method).

Photosynthesis in the shade (*A*_*500*_) depended on species, light treatment and their interaction (Fig. 7). Photosynthesis in the light (*A*_*1200*_) of leaves grown under full light depended on species (it was meaningless to test this on shade leaves hence the elimination of the shade treatment data). Forest species tended to have a lower photosynthesis than savanna species, except when the latter were grown in the shade (Fig. 7), in which case they were similar for both groups. Under full light, savanna species reached photosynthetic rates almost twice as large as those of forest species. Leaves of savanna species grown in the shade treatment tended to have a lower *A*_*500*_ than leaves grown in full light. Forest species showed the same *A*_*500*_ for leaves grown in shade and in full light.

*Ceiba* had a more erect stem than the other species, with a mean stem leaning ratio (*HL*) very close to 1 (Table 3 and Appendix 4, Fig. A4.1). *Cynometra* had a more erect stem than savanna species. *HL* was relatively constant for the forest species, while it decreased with age and increased under shade for savanna species, showing a tendency to spread rather than grow erect.

The number of axes *n*_*A*_ was similar for all species in the shade, but savanna species had greater values in full light and even more in the burnt × light treatment (Fig. A4.1).

**Figure 8:**
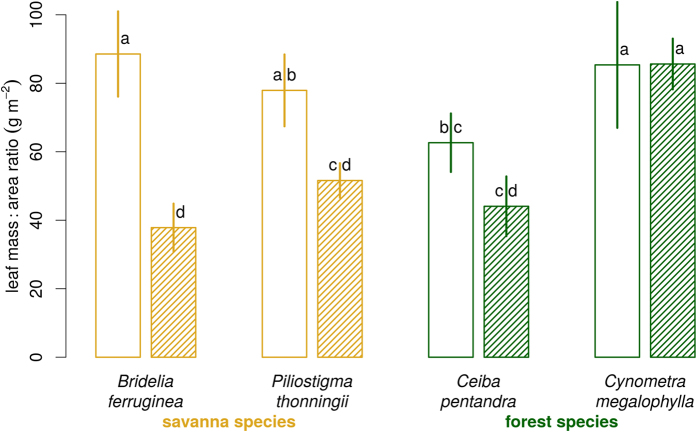
Leaf mass:area ratio *LMA*_*L*_ at age 6 months of seedlings grown in the open plot (open bars) or in the shaded plot (hashed bars). Error bars represent ± 1 standard deviation (n = 5). Bars with the same letter are not significantly different at the 5% level (goupings based on the Tukey multiple comparison test).

The leaf mass: area ratio at individual leaf level (*LMA*_*L*_) varied significantly with species and light treatment (Fig. 8). Forest species showed no significant change in *LMA*_*L*_ with light treatment. Savanna species adapted their *LMA*_*L*_ to the light regime (Fig. 8). The same pattern still held at date 9 when measured at the whole plant level, but not at date 15 after the dry season and the fire (*LMA*_*P*_, Fig. A4.1). Fire did not affect *LMA*_*P*_ except for *Ceiba* (Fig. A4.1).

## Discussion

Three of our study species were able to increase their initial biomass by two orders of magnitude over the time course of the experiment, while the fourth one (*Cynometra*) increased its biomass by only one order. Savanna species responded to the shading treatment, but not forest species, contrary to other studies in tropical rainforests manipulating much lower light levels^48^. What really made the difference between the two species groups was survival after topkill due to fire. Our savanna species did not suffer any overmortality due to fire, contrary to our forest species who showed a 30–50% increase of mortality in the fire treatment. Forest seedlings have been reported to have a much lower probability to survive fires compared to savanna seedlings^49^,^17^,^18^. Not only did our savanna species survive fire better than forest species, they also required ten times less biomass to resprout after fire. This size effect is not compensated by a bigger investment into root biomass: considering even the most extreme *rs* values of Fig. 5, surviving forest seedlings always had a higher root biomass than savanna species. This suggests that other aspects than biomass allocation are important to resist fire: investment into a thick bark^50^, non structural carbohydrate reserves^17^, or location of reserves – forest species might be storing reserves in their stem rather than in their roots. These results confirm that, for our study species, while fire is clearly the dominant factor shaping savanna seedling performance relative to forest species, competition for light is an important process to consider as it is present both in savanna and forest. They also show that looking at plant performance in response to treatments alone is not sufficient to understand the mechanisms involved.

If we follow our initial set of hypotheses (Table 1), where two ecosystems are characterized by three sets of constraints, one of them common to both, that rely on six different potential tradeoffs, we can improve our understanding by looking whether the study species behave as expected from their known growing habits: *Ceiba*, as a pioneer forest tree species, is expected to display traits mainly shaped by competition for light; *Cynometra*, a closed canopy understory tree species, is expected to display traits mainly shaped by shade tolerance; and the two savanna species are expected to display traits related to fire tolerance, but with possibly some impact of competition for light as a secondary constraint.

The first tradeoff concerned biomass allocation. It is particular in being a 3-poles tradeoff, i.e. a single pool of carbon assimilates is used to feed 3 different biomass compartments with different functional roles: leaves, the primary biomass factory; roots, used as reserves to resprout after a fire; and structural biomass, the key to win the race for light. Overall, our savanna species invested more in roots (33–43% vs. 23–35%, Table 4), had a higher *rs*, specially at small sizes, and a higher *ls* (Fig. 5). Forest species invested more into stems (38–58% vs. 22–30%, Table 4), and reached a lower *ls.* This conforms to the hypotheses of Table 1 and to literature results (Fig. 6). *rs* tends to be higher in savannas than in forests *on average*^18^,^46^,^51^. Species from humid savannas (with more intense and more frequent fires) invest more into roots than species of dry savannas^43^. The surprise was that our savanna species invested more into leaves (28–40%, Table 4) than the shade tolerant species (25%). In a fire-prone environment, seedlings have a limited time to build up their belowground reserves before the first fire^18^,^52^. For savanna seedlings ‘expecting’ a fire sooner or later, it is important to build a belowground storage system *quickly*. Our results suggest that this requires investing into leaves first, then roots (or any belowground storage organs), at the detriment of stems.

The second tradeoff was about trunk or main stem shape. With the same biomass allocated to the main stem, it is possible to construct a more conical or more cylindrical stem. A cylindrical stem (thinner) is the best way to quickly reach a tall height, whereas a conical shape (thicker) is a way to reduce stem biomass loss through topkill in fires. Here, *Ceiba* grows the tallest *H*_*max*_, behaving as expected for a dominant forest species, followed by *Cynometra* and the savanna species. However, the same hierarchy applies to *D*_*max*_ and, in the end, the *LD* ratio, that measures stem taper (the larger the ratio, the finer the stem), is smaller for *Ceiba* and similar for other species. This was unexpected from Table 1.

The third tradeoff is physiological: it is not possible for a plant to be able to survive in dense shade and to grow fast. In general, shade tolerance is associated with a lower light compensation point (light level at which carbon gain through photosynthesis exactly compensates losses by respiration), at the cost of a lower maximal assimilation rate *A*_*max*_^53^. This is easily explained by the selective pressure the heavy shade of closed canopy forests (down to 1–2% of full light) casts on small seedlings: survival is more important than growth in such environments, and apparently the only way to respond is to decrease the compensation point^54^,^55^. As a consequence, species with a low compensation point reach a lower *A*_*max*_ at a lower light level than more light demanding species. Although we did not put our seedlings at shade levels as low as those encountered in forests, their responses are consistent: our forest species have developed some shade tolerance relative to our savanna species, at the cost of a lower maximal leaf assimilation rate in high light *A*_*1200*_, but this maximal rate is reached in mild shading so that *A*_*500*_≈*A*_*1200*_ This pattern has been observed between shade tolerant and pioneer rainforest species^46^,^56^,^57^, and between co-occurring savanna and forest species^26^. Photosynthetic rates of *Ceiba* were consistent with those reported in Central America^58^. Cerrado species exhibit the same reaction to shading as our savanna species^59^. All results on size variables and relative growth rate confirmed this pattern: the savanna species showed a strong decrease in size and growth when in shade, whereas the forest species seemed unaffected by the light level. A similar difference, but between shade tolerant and pioneer forest species, was found by Agyeman *et al*.^60^.

The fourth tradeoff is another morphological tradeoff: a stem cannot be both erect and prostrate. In the context of competition for light, a slight difference in investment into height at the seedling stage can make the difference between a dominant and a dominated tree at an early stage in the development, and result in death or survival. In the context of fire, buds above flame height (~2 m in our case) have a much better chance of survival. While *Ceiba* has an erect orthotropic main stem, the other three species have an orthotropic-plagiotropic main axis (erect near the ground and bending over to become more horizontal towards the top – comparable to the Troll architectural model^61^). The *HL* ratios confirmed this, with *Ceiba* behaving as expected for a pioneer forest species with a very straight, erect stem (*HL*≈1), and savanna species having more leaning stems. The surprise came from *Cynometra*, which presented an intermediate leaning. Investment into stem is not a priority for savanna species, suggesting another factor than competition for light is driving their allocation strategy.

The fifth tradeoff states that a plant can distribute its stem biomass into a variable number of stems. The need to expose leaves optimally to light may cause a higher branching. Our species behaved as expected, with savanna species having the greatest number of ramifications in full light. Apparently, fire also caused a further increase in ramification, a phenomenon already observed in connection with reserve dynamics^62^.

Finally, there is apparently a link between leaf mass area and shade tolerance: dense shade favours thinner leaves, i.e. with a lower *LMA*. The savanna species responded in this way, by reducing their *LMA*_*L*_ and *LMA*_*P*_ in shade compared to full light. The forest species did not show such a trend. *Cynometra*, the most shade tolerant species, had the highest *LMA*, possibly because of its evergreen leaves^63^.

Our experiment was meant to test the effects of shade, the main factor driving forest dynamics^64^, and that of fire, the main factor driving humid savannas, on tree seedlings of species coming from these two ecosystems. Despite a few surprises, our results are well in accordance with the expectations of Table 1 based on previous knowledge of tradeoffs between traits related to shade tolerance, competition for light in the open, and fire tolerance. Our understorey forest species, *Cynometra megallophylla*, has traits compatible with those of a shade tolerant species: biomass allocation to leaves higher than in *Ceiba*; a low *RGR* that does not improve in full light; a maximal assimilation already saturated at the two light levels tested here (mild shade and full light); a rather prostrate bearing. Our supposed pioneer species, *Ceiba pentandra*, has traits compatible with this group: a high allocation to stems; a straight erect stem; an assimilation reacting in similar ways as *Cynometra* in full light and mild shade; a lower *LMA* than savanna species in full light. The two savanna species, *Bridelia ferruginea* and *Piliostigma thonningii*, had very similar responses, with traits compatible with frequent fires: a high investment into roots, but also in leaves probably to produce those roots quickly; a maximal assimilation characteristic of light demanding species, increasing in full light in our experimental conditions. The picture was not 100% as expected though. Savanna species had the most prostrate bearing, which is unexpected under high competition for light and high fire frequency; it may be due to their prioritary investment into leaves and roots, that leaves very little biomass for stems. *Ceiba* had the most conical stem, where we expected the most cylindrical stem; it may be due to its buttress-trunk habit, well-known at the adult stage; or to a misconception of what is required to build up a self-sustained orthotropic shoot. *Cynometra* had an intermediate leaning. Finally, survival to fire depended only on seedling size, not on investment into roots; but the size required to survive fire was ten times bigger in forest species than in savanna species.

Plant traits are the result of multiple constraints and, as the pattern and process problem states it, there is no one-to-one relationship between a trait (=pattern) and a constraint (=process) that caused its evolution. In the particular case of this study, the initially apparently simple question – is there a tradeoff between fire resistance and growth under competition for light that may explain differences observed between co-occurring savanna and forest species – was too naive to be addressed directly. We had to realize that there were more constraints (3) than ecosystems (2), and that the ‘global’ tradeoff was actually made of six ‘elementary’ tradeoffs. A tradeoff is often understood as any negative relationship between two traits; we believe, and our results support it, that a tradeoff has to be also underpinned by a physical or biological constraint linking these two variables. For example, there is a tradeoff between photosynthesis compensation point and maximal assimilation because of the limitations of plant physiology; a plant cannot be both prostrate and erect; total assimilated biomass must be distributed among various compartments; etc. Only at this level can we affirm that there is a tradeoff; the supposed negative relation between ‘growth under competition for light’ and ‘fire resistance’ then arises as a complex combination of these elementary tradeoffs. If we do not go down to the ultimate limitations of elementary tradeoffs, we cannot understand why a plant should not be a good competitor for light *and* fire tolerant. Prior to conclude on ecotone future, one has to really identify which sets of constraints, associated elementary tradeoffs, and biological traits, are required to understand the behaviour of the plant species, as proposed and examplified in this study.

From our findings, we come up with questions for further work. Although, from the literature, investment into roots is important for fire survival, it is clearly not enough. What other traits could explain that a forest tree seedling has to be ten times bigger than a savanna tree seedling to resprout after topkill? Where are reserves in tree seedlings? Do savanna tree species have a high compensation point of their light-photosynthesis response curve? Is it the reason of their exclusion at the seedling stage from the forest understory?

## Additional Information

**How to cite this article**: Gignoux, J. *et al*. Allocation strategies of savanna and forest tree seedlings in response to fire and shading: outcomes of a field experiment. *Sci. Rep.* **6**, 38838; doi: 10.1038/srep38838 (2016).

**Publisher's note:** Springer Nature remains neutral with regard to jurisdictional claims in published maps and institutional affiliations.

## Supplementary Material

Supplementary Appendices

## Figures and Tables

**Table 1:** Expected tradeoffs resulting from responses of plants to environmental constraints encountered in savannas and forests, and the associated relevant traits to measure on plants.

Tradeoff on	Main environmental constraint			Relevant traits
	Dense shade	Competition for light in the open	Frequent fires	
Biomass allocation	to *leaves* to optimize light capture^46^,^65^	to *stems & leaves* to optimize height and shading	to *belowground reserves* to resprout after topkill^18^,^43^	Biomass ratios: % root, % leaves and % stem biomass; Biomass allocation coefficients: biomass ratios per unit time
Trunk shape	NA	*cylindrical* to maximize height at a low biomass cost^30^	*conical* to decrease stem ignition risk by having a thick stem and reduce biomass loss through topkill^30^	Stem length:basal diameter ratio
Photo-synthesis	low *compensation point* to maximize survival in low light^53^	high *maximal assimilation rate* to maximize growth rate^53^	NA	Maximal assimilation rate as a function of light irradiance
Trunk bearing	*prostrate* to reduce self shading^65^,^66^	*erect* to optimize height and reduce excessive irradiation^67^	*erect* to reduce fire damage^40^	Stem height:length ratio
Ramification	NA	*low*, priority to height	*high*, priority to light capture	Number of branches, branch biomass
Leaf morphology	Thin leaves^41^	Thick leaves^41^	NA	Leaf mass:area ratio
*Ecosystem*	*Forest*			
		*Savanna*		

NA, not applicable.

**Table 2:** Spearman correlation coefficients (*r*) between all trait variables (Fig. 1).

	*W*	*W*_*abg*_	*W*_*R*_	*W*_*str*_	*W*_*L*_	*D*_*max*_	*L*_*P*_	*H*_*max*_	*V*_*p*_	*n*_*A*_	*n*_*L*_	*LA*	*ls*	*rs*	*HL*	*LD*	*LMA*_*P*_	*RGR*
*W*	**1.00**	**0.99**	**0.98**	**0.97**	**0.94**	**0.85**	**0.90**	**0.85**	**0.90**	0.52	**0.78**	**0.90**	−0.37	−0.25	0.33	−0.41	*0.07*	0.17
*W*_*abg*_		**1.00**	**0.95**	**0.98**	**0.94**	**0.87**	**0.91**	**0.87**	**0.92**	0.48	**0.76**	**0.89**	−0.42	−0.35	0.35	−0.43	*0.06*	0.17
*W*_*R*_			**1.00**	**0.91**	**0.92**	**0.76**	**0.87**	**0.78**	**0.81**	0.56	**0.77**	**0.86**	−0.28	*−0.08*	0.28	−0.36	*0.10*	0.16
*W*_*str*_				**1.00**	**0.88**	**0.90**	**0.88**	**0.91**	**0.94**	0.37	0.67	**0.81**	−0.56	−0.41	0.45	−0.47	*0.06*	*0.10*
*W*_*L*_					**1.00**	**0.73**	**0.88**	**0.75**	**0.79**	0.66	**0.89**	**0.98**	−0.15	−0.20	0.13	−0.29	*0.08*	0.28
*D*_*max*_						**1.00**	**0.84**	**0.91**	**0.99**	0.23	0.60	0.73	−0.62	−0.69	0.61	−0.65	*0.01*	0.11
*L*_*P*_							**1.00**	**0.90**	**0.91**	0.45	**0.80**	**0.83**	−0.35	−0.26	0.31	−0.27	*−0.01*	0.21
*H*_*max*_								**1.00**	**0.94**	0.29	0.67	0.68	−0.63	−0.43	0.61	−0.42	*−0.02*	0.19
*V*_*p*_									**1.00**	0.26	0.64	**0.79**	−0.59	−0.69	0.59	−0.55	*−0.01*	0.12
*n*_*A*_										**1.00**	0.67	0.59	0.33	0.21	−0.16	−0.16	*0.11*	0.12
*n*_*L*_											**1.00**	**0.85**	*0.11*	*−0.04*	0.12	−0.28	*0.02*	0.40
*LA*												**1.00**	*−0.12*	−0.47	0.16	−0.27	*−0.12*	0.42
*ls*													**1.00**	0.53	−0.73	0.43	*−0.03*	0.29
*rs*														**1.00**	−0.44	0.39	*0.05*	*−0.08*
*HL*															**1.00**	−0.44	*−0.01*	*0.05*
*LD*																**1.00**	−0.14	*0.01*
*LMA*_*P*_																	**1.00**	−0.32
*RGR*																		**1.00**

*Italics* indicate non significant correlation coefficients (approximate t-tests). **Bold** indicate high correlation coefficients (|*r*| ≥ 0.75).

**Table 3:** Significance of fixed effects and contrast tests in analyses of variances of the effects of ecosystem (forest vs savanna), species within ecosystem (*Ceiba* and *Cynometra* within forest, *Bridelia* and *Piliostigma* within savanna), shading (shade vs light), and fire (burnt vs protected) on all raw plant size variables (Fig. 1) and allometric ratios.

*fixed effects (F tests)*	*size variables*												*ratios*					*RGR*
	*W*	*W*_*abg*_	*W*_*R*_	*W*_*str*_	*W*_*L*_	*LA*	*L*_*P*_	*n*_*L*_	*H*_*max*_	*n*_*A*_	*D*_*max*_	*V*_*P*_	*HL*	*LD*	*LMA*_*P*_	*rs*	*ls*	
(Intercept)	***	***	***	***	***	***	***	***	***	***	***	***	***	***	***	***	***	***
treatment	***	***	***	***	***	***	***	***	***	***	***	***	***	***	***	***	***	***
age	—	—	—	—	—	—		**	***	***	—	—	***	—	—	—	—	—
spatial effects	***	***	**	**	***	***		*		*	**	**				**	.	**
***contrasts for treatment (t tests)***																		
forest – savanna		**		***	.	***	***	***	***	***	***	***	***	***		***	***	***
*Ceiba*: 3 – 9	***	***	***	***	***	—	—	—	—	—	—	—	—	—	—	***	**	***
*Ceiba*: 9 – 15	***	***	***	***	*	.	—	—	—	—	***	***	—		***	**	***	***
*Cynometra*: 3 – 9	***	***	***	***	***	—	—	—	—	—	—	—	—	—	—		*	***
*Cynometra*: 9 – 15	**	*	***	*	.		—	—	—	—	—	—	—	—			.	
*Bridelia*: 3 – 9	***	***	***	***	***	—	—	—	—	—	—	—	—	—	—	*		***
*Bridelia*: 9 – 15	***	***	***	***	***	*	—	—	—	—	***	***	—	*	.			***
*Piliostigma*: 3 – 9	***	***	***	***	***	—	—	—	—	—	—	—	—	—	—	***		***
*Piliostigma*: 9 – 15	***	***	***	***	***	***	—	—	—	—	***	***	—		**	***		***
forest:shading							**	**	**	**			***				***	
savanna:shading	***	***	***	***	***	***	***	***	***	***	***	***		**	***	***		***
forest:fire							***	**	***	***	—	—	*	—		*		*
savanna:fire		.	.				***	***	**	***	***	*	***	*				
light: *Ceiba* – *Cynometra*	***	***	*	***		**				*	***	***		.	***	***	***	***
light: *Bridelia* – *Piliostigma*	**	**	*	*	**		***	***	***	***			***			*		
shade: *Ceiba* – *Cynometra*	***	***	*	***	**	***	*	*	***	***	***	***	***	**	***	***	***	***
shade: *Bridelia* – *Piliostigma*							***	***	***	***			***		.	***		
burnt: *Ceiba* – *Cynometra*	**	**	**	**	***	***	***	*	***	***	—	—	*	—		*	*	
burnt: *Bridelia* – *Piliostigma*							***	***	.	***	—	—	***	—				
protected: *Ceiba* – *Cynometra*	—	—	—	—	—	—	.	.	.	***	—	—		—	—	—	—	—
protected: *Bridelia* – *Piliostigma*	—	—	—	—	—	—	***	***	***	***	—	—	***	—	—	—	—	—

Non-destructive variables (*L*_*P*_*, n*_*L*_*, H*_*max,*_ *n*_*A*_*, D*_*max*_*, V*_*P*_, *HL, LD*) were analysed through linear mixed models, all other variables through simple linear models. All variables were log-transformedbefore analysis. Date was treated as a covariate for some variables, and as a factor for others, depending on thenumber of measurement dates for each variable (see Appendix 4 for details of statistical models and contrast tests). Variables named as in Fig. 1. —: term not present in specific model; ***: P < 0.001; **: 0.001 ≤ P < 0.01; *: 0.01 ≤ P < 0.05;. : 0.05 ≤ P < 0.1.

**Table 4:** Allocation coefficients to leaf biomass, structural biomass, and root biomass for the studied species according to the shading treatment at date 9.

Species	Biomass compartment	shading		Final model r^2^
		L	S	
*Bridelia ferruginea*	leaf	0.359	0.283	0.995
	stem	0.308	0.258	0.986
	root	0.330	0.432	0.993
	*sum*	*0.997*	*0.973*	
*Ceiba pentandra*	leaf	0.177	0.177	0.942
	stem	0.575	0.575	0.989
	root	0.263	0.236	0.994
	*sum*	*1.015*	*0.988*	
*Cynometra megalophylla*	leaf	0.249	0.249	0.987
	stem	0.386	0.386	0.995
	root	0.349	0.349	0.990
	*sum*	*0.984*	*0.984*	
*Piliostigma thonningii*	leaf	0.396	0.275	0.989
	stem	0.266	0.219	0.993
	root	0.437	0.356	0.994
	*sum*	*1.099*	*0.850*	

Shading, L (light) and S (shade). Coefficients computed as slopes of regressions (forced through the origin) between increase in biomass compartments and increase in total biomass between dates 3 and 9. All coefficients are significant at the 5% level. Regression models were simplified until all remaining effects were significantly different from each other (equal coefficients were merged into a single coefficient). The sum of the three coefficients should be equal to 1, however since they were estimated as regression coefficients there was no such constraint put on their estimation: we provide the sum as an assessment of the estimate quality.
