# Supplementary material for: Allocation strategies of savanna and forest tree seedlings in response to fire and shading: outcomes of a field experiment
Source: Sci Rep. 2016 Dec 21;6:38838. doi: 10.1038/srep38838 (PMC5175285; doi:10.1038/srep38838)
Supplement: Supplementary Appendices [file srep38838-s1.pdf]

# Allocation strategies of savanna and forest tree seedlings in response to fire and shading: outcomes of a field experiment

Jacques Gignoux, Souleymane Konaté, Gaëlle Lahoreau, Xavier Le Roux, Guillaume Simioni

# Appendices

# Appendix 1. Weather data

**Fig. A1.1** 44-year average of monthly rainfall (bars) and monthly mean (solid line), min and max (dashed lines) temperatures. Meteorological data from the Lamto Geophysical Station. Error bars for rainfall represent one standard deviation.

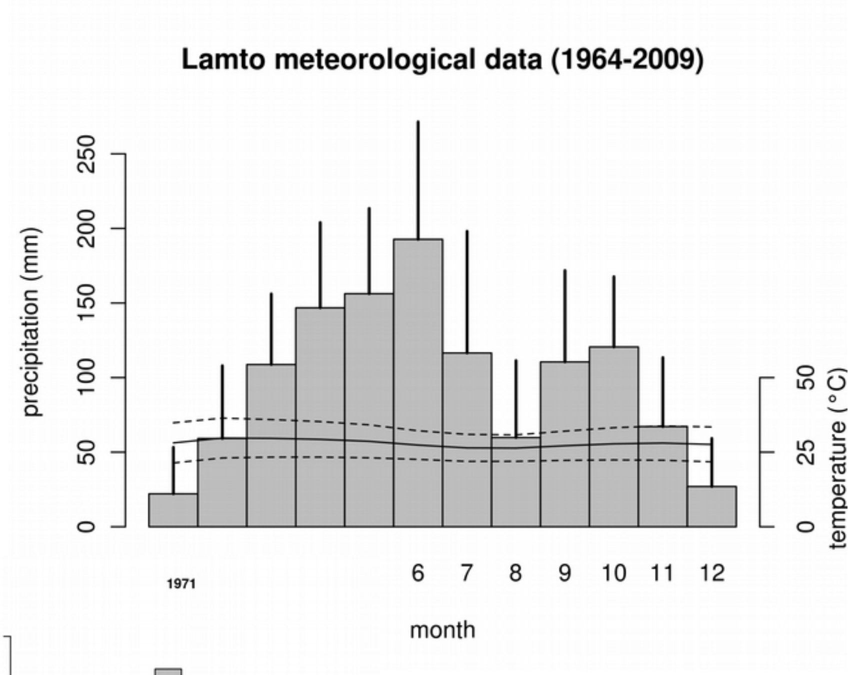


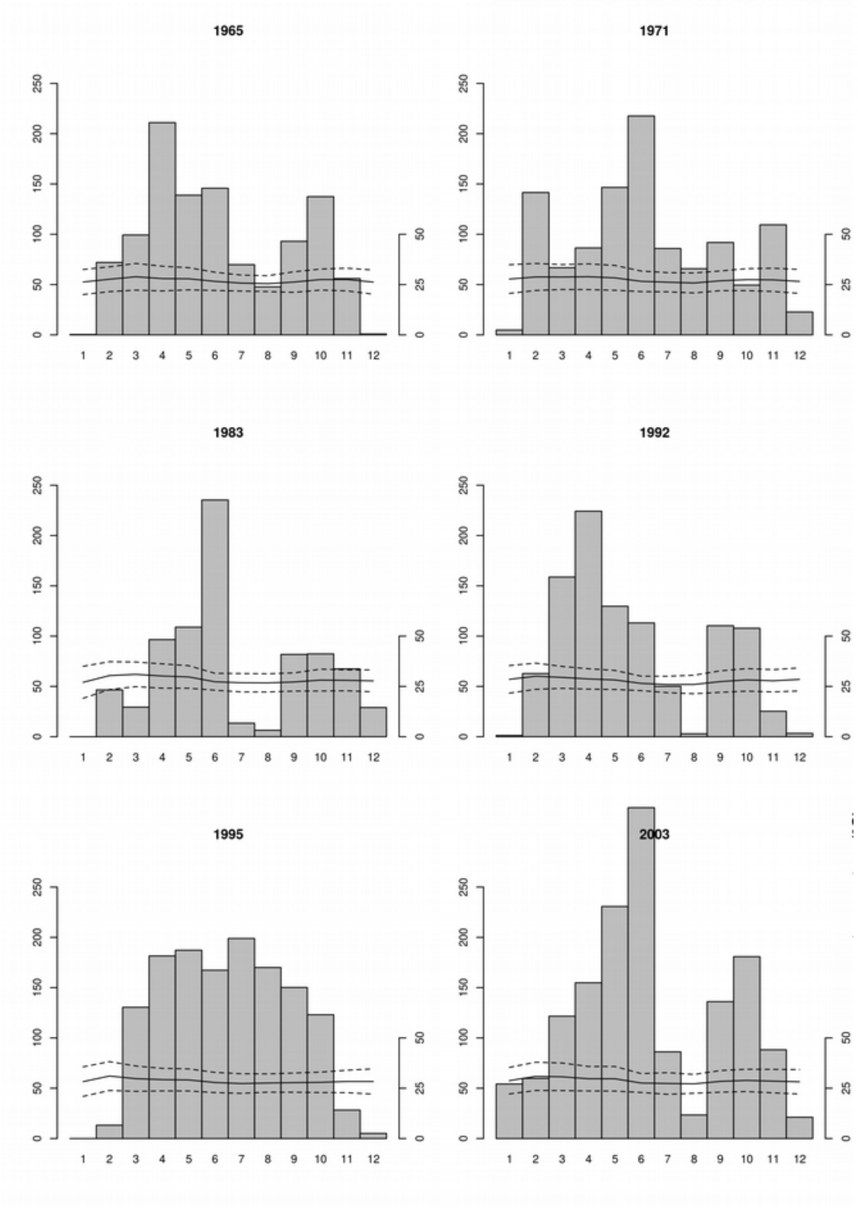


**Fig. A1.2** Six particular years extracted from the metorological data to illustrate the year-to-year variability in total rainfall, date and duration of the dry seasons : 1965 and 1992 represent 'normal' years with a marked short dry season in August and two very dry months of long dry season, 1971 and 1995 are years where the short dry season did not happen, 1983 is a particularly dry year, and 2003 is a particularly wet year with almost no long dry season in January.  Bars and lines as in fig. A1.1.

# Appendix 2. Estimate of biomasses from non-destructive measurements

Biomass variables (*W*, $W_{abg}$, $W_R$, $W_{str}$, $W_l$) were destructively measured on 286 seedlings (Fig. 1). In order to compute relative growth rate (*RGR*) and allocation coefficients ($w'_L$, $w'_{str}$, $w'_R$), we fitted regression functions to predict those variables from non-destructive measurements. These regressions were then used to predict biomass variables at ages 3, 9 and 15 for all seedlings. The following non-destructive measures were used as predictors: $H_{max}$, $n_L$, $n_B$, *L*, $L_P$, $n_S$, $n_A$. We did not use $D_{max}$ or $V_P$ because these variables were not measured at all sampling dates. Among the numerous predictions, we retained thosed based only on $n_L$ and $L_P$, as a best compromise between prediction accuracy and simplicity of the models: predictions based on different predictors for each species would have been slightly more precise, but we found untractable to handle predictor variables specific to each [*species * predicted variable * date*] combination. There was a significant date effect on most relations (Table A2.1). We checked the quality of these predictions by plotting predicted vs. observed biomass variables (Figure A2.1).

| ***Predicted variable*** | ***species*** | ***date*** | ***intercept*** | ***slope for $n_L$*** | ***slope for $L_P$*** | ***$R^2$*** |
|---|---|---|---|---|---|---|
| $\hat{W}_{abg}$ | *Bridelia ferruginea* | 3 | -10.646 | 1.475 | 2.012 | 0.966 |
| | | 9 | -7.258 | 0.933 | 1.566 | 0.938 |
| | | 15 | -3.448 | 1.207 | 0.214 | 0.943 |
| | *Ceiba pentandra* | 3 | -7.971 | -0.380 | 2.635 | 0.861 |
| | | 9 | -3.802 | 0.741 | 1.152 | 0.885 |
| | | 15 | -8.800 | 0.295 | 2.590 | 0.972 |
| | *Cynometra megalophylla* | 3 | -4.992 | 0.659 | 1.321 | 0.920 |
| | | 9 | -2.924 | 0.542 | 0.967 | 0.736 |
| | | 15 | -4.588 | 0.225 | 1.633 | 0.876 |
| | *Piliostigma thonningii* | 3 | -5.044 | 0.082 | 1.048 | 0.300 |
| | | 9 | -4.712 | 0.410 | 1.442 | 0.696 |
| | | 15 | -2.955 | 0.963 | 0.573 | 0.750 |
| $\hat{W}_L$ | *Bridelia ferruginea* | 3 | -10.853 | 1.610 | 1.829 | 0.883 |
| | | 9 | -6.484 | 1.060 | 1.123 | 0.959 |
| | | 15 | -3.770 | 1.180 | 0.193 | 0.962 |
| | *Ceiba pentandra* | 3 | -4.951 | 1.093 | 0.527 | 0.421 |
| | | 9 | -5.504 | 0.697 | 1.235 | 0.965 |
| | | 15 | -8.222 | 0.745 | 1.738 | 0.908 |
| | *Cynometra megalophylla* | 3 | -6.050 | 0.690 | 1.346 | 0.908 |
| | | 9 | -3.730 | 0.584 | 0.906 | 0.864 |
| | | 15 | -6.033 | 0.272 | 1.697 | 0.909 |
| | *Piliostigma thonningii* | 3 | -5.906 | 0.361 | 1.062 | 0.213 |
| | | 9 | -6.116 | 0.638 | 1.439 | 0.923 |
| | | 15 | -3.251 | 0.961 | 0.504 | 0.754 |

| | | | | | | |
|---|---|---|---|---|---|---|
| $\hat{W}_R$ | *Bridelia ferruginea* | 3 | -8.799 | 1.683 | 1.160 | 0.710 |
| | | 9 | -6.089 | 0.610 | 1.507 | 0.873 |
| | | 15 | -2.507 | 0.903 | 0.265 | 0.935 |
| | *Ceiba pentandra* | 3 | -9.479 | -1.630 | 3.449 | 0.839 |
| | | 9 | -3.620 | 0.584 | 1.013 | 0.718 |
| | | 15 | -4.975 | 0.492 | 1.491 | 0.910 |
| | *Cynometra megalophylla* | 3 | 0.179 | 1.121 | -0.497 | 0.688 |
| | | 9 | -0.946 | 0.496 | 0.366 | 0.501 |
| | | 15 | -2.670 | 0.228 | 1.063 | 0.787 |
| | *Piliostigma thonningii* | 3 | -7.172 | 0.155 | 1.456 | 0.365 |
| | | 9 | -3.396 | -0.166 | 1.543 | 0.611 |
| | | 15 | -1.302 | 0.652 | 0.521 | 0.716 |
| $\hat{W}_{str}$ | *Bridelia ferruginea* | 3 | -12.296 | 1.303 | 2.341 | 0.978 |
| | | 9 | -10.281 | 0.798 | 2.202 | 0.897 |
| | | 15 | -4.728 | 1.255 | 0.240 | 0.915 |
| | *Ceiba pentandra* | 3 | -10.680 | -1.183 | 3.781 | 0.879 |
| | | 9 | -4.054 | 0.748 | 1.142 | 0.833 |
| | | 15 | -9.877 | 0.183 | 2.858 | 0.962 |
| | *Cynometra megalophylla* | 3 | -5.392 | 0.634 | 1.300 | 0.919 |
| | | 9 | -3.579 | 0.528 | 1.011 | 0.558 |
| | | 15 | -4.907 | 0.194 | 1.625 | 0.850 |
| | *Piliostigma thonningii* | 3 | -5.645 | -0.344 | 1.096 | 0.551 |
| | | 9 | -5.665 | 0.397 | 1.474 | 0.596 |
| | | 15 | -4.163 | 0.958 | 0.668 | 0.739 |
| $\hat{W}$ | *Bridelia ferruginea* | 3 | -9.250 | 1.544 | 1.688 | 0.903 |
| | | 9 | -5.844 | 0.816 | 1.473 | 0.940 |
| | | 15 | -2.215 | 1.074 | 0.217 | 0.948 |
| | *Ceiba pentandra* | 3 | -7.760 | -0.635 | 2.798 | 0.856 |
| | | 9 | -3.127 | 0.678 | 1.123 | 0.868 |
| | | 15 | -6.803 | 0.362 | 2.206 | 0.961 |
| | *Cynometra megalophylla* | 3 | -1.343 | 0.931 | 0.313 | 0.867 |
| | | 9 | -1.505 | 0.517 | 0.746 | 0.730 |
| | | 15 | -2.908 | 0.230 | 1.347 | 0.862 |
| | *Piliostigma thonningii* | 3 | -5.026 | 0.120 | 1.148 | 0.332 |
| | | 9 | -3.211 | 0.138 | 1.462 | 0.687 |
| | | 15 | -1.316 | 0.802 | 0.533 | 0.743 |

**Table A2.1**. Predictions of variables obtained through destructive measurements ('Predicted variable') from variables obtained through non-destructive measurements (number of leaves and total stem length). Results of multiple linear regressions on log-transformed predicted and predictor variables. Dates in months from the beginning of the experiment.

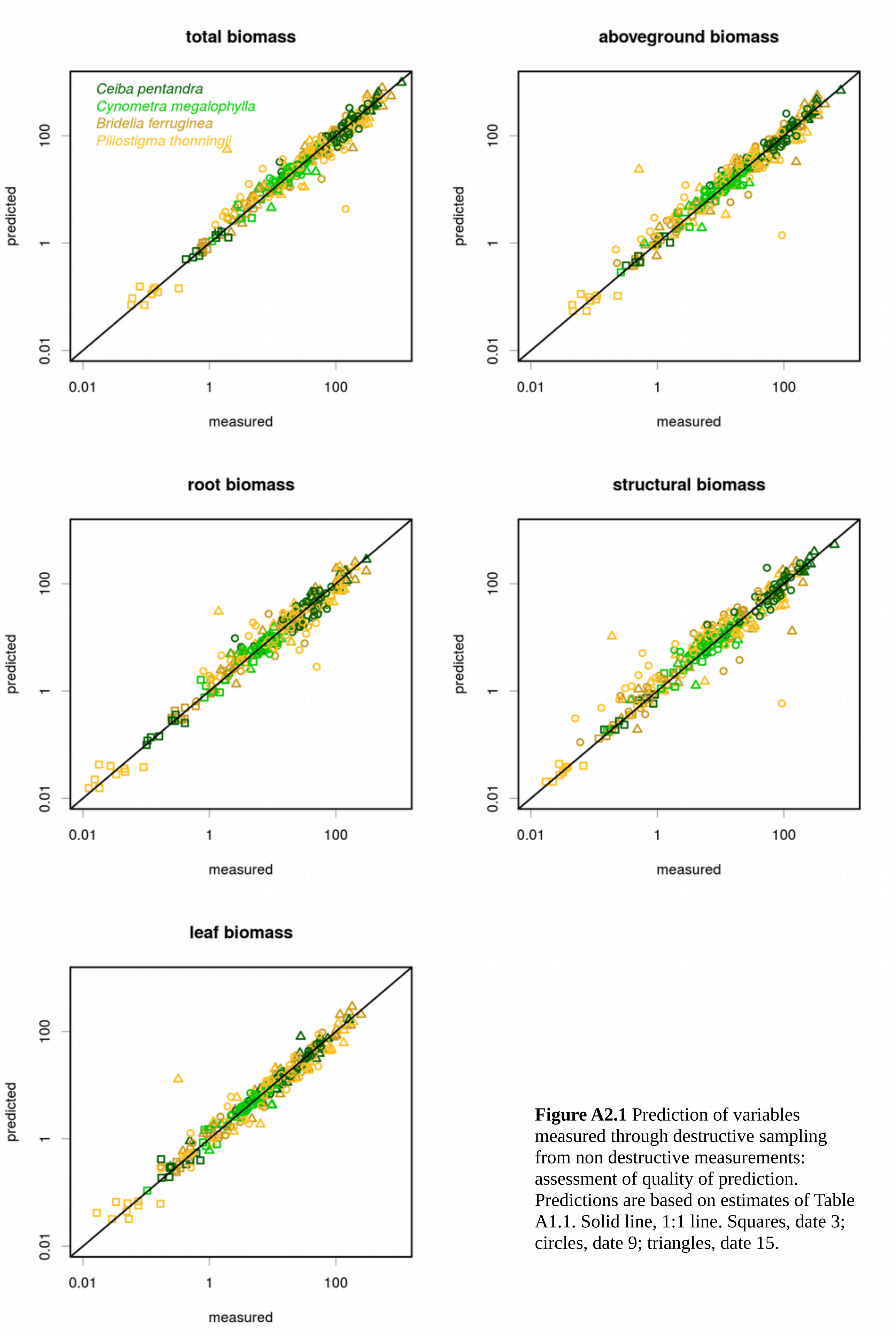


**Figure A2.1** Prediction of variables measured through destructive sampling from non destructive measurements: assessment of quality of prediction. Predictions are based on estimates of Table A1.1. Solid line, 1:1 line. Squares, date 3; circles, date 9; triangles, date 15.

# Appendix 3. Seed mass of the study species

We used literature estimates of seed mass of our study species to estimate the seedling biomass at time 0, in order to compute their relative growth rate (*RGR*) at age 3.

| ***species*** | ***seed mass (mg)*** | ***Source*** |
|---|---|---|
| *Bridelia ferruginea* | 47.8 | Lahoreau et al. 2006 |
| *Ceiba pentandra* | 116.0 | Allen et al. 2003 |
| *Cynometra megalophylla* | 5900.0 | Hovestadt et al. 1999 |
| *Piliostigma thonningii* | 95.9 | Lahoreau et al. 2006 |

**Table A3.1**. Seed mass of the study species.

**Data sources:**


Allen E.B., Allen M.F., Egerton-Warburton L., Corkidi L. & Gomez-Pompa A., 2003. Impacts of early- and late-seral mycorrhizae during restoration in seasonal tropical forest, Mexico? *Ecological Applications,* 13(6):1701-1717.

Lahoreau G., Barot S., Gignoux J., Hoffmann W.A., Setterfield S.A. & Williams P.R., 2006. Positive effect of seed size on seedling survival in fire-prone savannas of Australia, Brazil and West Africa. *Journal of Tropical Ecology* 22:719-722.

Hovestadt T., Yao P. & Linsenmair K.E., 1999. Seed dispersal mechanisms and the vegetation of forest islands in a West African forest-savanna mosaic (Comoé National Park, Ivory Coast). *Plant Ecology*, 144:1-25.

# Appendix 4. Statistical methods used for analysis of variance of size and trait variables

**Statistical design –** The statistical design of our experiment for the variables $L_P$, $n_L$, $H_{max}$, $n_A$, $D_{max}$, $V_P$, $W$, $W_{abg}$, $W_R$, $W_{str}$, $W_L$, $LA$, $LD$, $HL$, $LMA_P$, $RGR$ (Fig. 1) was as follows :

- there were four study species, 2 forest and 2 savanna species

- seedlings were transplanted at age 3 months from the nursery to the experimental plots (4 blocks)

- from age 3 to the end of the experiment, seedlings were subject to a shading treatment with two levels: shade or full light

- at time 10 months, fire was set to half of the plots, so that the fire treatment only applied to seedlings at age 15.

Seedlings were sampled destructively at age 3 (seedlings directly taken from the nursery), 9 and 15, so that the number of available seedlings for non-destructive measurements decreased along the course of the experiment (see Fig. 1 for the list of destructive and non-destructive measurements). Numbers of seedlings available for non-destructive and destructive measurements are given in tables A4.1 and A4.2

| age | treatments | | block | ecosystem / species | | | | total per date | total per treatment | |
|---|---|---|---|---|---|---|---|---|---|---|
| | | | | Forest | | Savanna | | | | |
| | shading | fire | | CEI | CYN | BRI | PIL | | | |
| 3 | *nursery seedlings* | | | **41** | **41** | **42** | **32** | 156 | | |
| 3 | - | - | 1 | **27** | **27** | **27** | **30** | 111 | | |
| 5 | L | - | 1 | **27** | **27** | **27** | **27** | 108 | | |
| 9 | L | - | 1 | **27** | **25** | **27** | **27** | 106 | | 280 |
| 15 | L | P | 1 | **18** | **12** | **18** | **18** | 66 | 66 | |
| 3 | - | - | 2 | **28** | **28** | **27** | **29** | 112 | | |
| 5 | O | - | 2 | **27** | **27** | **27** | **27** | 108 | | |
| 9 | O | - | 2 | **27** | **27** | **27** | **27** | 108 | | 287 |
| 15 | O | P | 2 | **19** | **15** | **18** | **19** | 71 | 71 | |
| 3 | - | - | 3 | **27** | **27** | **27** | **28** | 109 | | |
| 5 | L | - | 3 | **27** | **27** | **27** | **27** | 108 | | |
| 9 | L | - | 3 | **27** | **27** | **27** | **27** | 108 | | 263 |
| 15 | L | B | 3 | **9** | **1** | **19** | **18** | 47 | 47 | |
| 3 | - | - | 4 | **27** | **27** | **27** | **31** | 112 | | |
| 5 | O | - | 4 | **27** | **26** | **27** | **27** | 107 | | |
| 9 | O | - | 4 | **27** | **26** | **27** | **27** | 107 | | 261 |
| 15 | O | B | 4 | **8** | **2** | **16** | **21** | 47 | 47 | |
| total per species | | | | 420 | 392 | 437 | 442 | 1691 | 231 | 1091 |
| total per ecosystem | | | | 812 | | 879 | | | | |

**Table A4.1**. Numbers of available data for the non-destructive measurements (Fig.1). $L_P$, $n_L$, $H_{max}$, $n_A$ (hence $HL$) were measured at ages 3, 5, 9, 15. $D_{max}$, (hence $V_P$ and $LD$) was measured at ages 9 and 15. L, light; O, shade; B, burned; P, protected from fire. CEI, *Ceiba pentandra*; CYN, *Cynometra megalophylla*; BRI, *Bridelia ferruginea*; PIL, *Piliostigma thonningii*.

| age | treatments | | block | ecosystem / species | | | | total per date |
|---|---|---|---|---|---|---|---|---|
| | | | | Forest | | Savanna | | |
| | shading | fire | | CEI | CYN | BRI | PIL | |
| 3 | *nursery seedlings* | | | **9** | **9** | **9** | **9** | 36 |
| 9 | L | - | 1 | **9** | **9** | **9** | **9** | 36 |
| 15 | L | P | 1 | **9** | **8** | **9** | **10** | 36 |
| 9 | O | - | 2 | **9** | **8** | **9** | **9** | 35 |
| 15 | O | P | 2 | **-*** | **9** | **8** | **8** | 25 |
| 9 | L | - | 3 | **9** | **9** | **9** | **9** | 36 |
| 15 | L | B | 3 | **7** | **0** | **9** | **9** | 25 |
| 9 | O | - | 4 | **9** | **9** | **9** | **9** | 36 |
| 15 | O | B | 4 | **-*** | **2** | **9** | **10** | 21 |
| total per species | | | | 61 | 63 | 80 | 82 | 286 |
| total per ecosystem | | | | 124 | | 162 | | |

* The *Ceiba* seedlings grown under shade and protected from fire did so well that they reached the shading cloth; they were discarded from the dataset since this influenced their growth.

**Table A4.2**. Numbers of available data for the destructive measurements (Fig.1). $W$, $W_{abg}$, $W_R$, $W_{str}$, $W_L$, were sampled at ages 3, 9 and 15. *LA* (hence $LMA_P$) was sampled at ages 9 and 15. Codes as in table A4.1.

**Statistical models** – Since the design was not standard, we used  a 'flat' factor *f* combining ecosystem, species, and fire treatment (24 levels). All variables were log-transformed in analyses to stabilize the variance.

For *non-destructive variables* ($L_P$, $n_L$, $H_{max}$, $n_A$), a mixed linear model was fitted with the following fixed effects :

$$\mathrm{Log}(Y_{ijk}) = f_i + \alpha \mathrm{Log}(age) + (\beta x + \gamma y + \delta xy + \zeta x^2 + \eta y^2) + \varepsilon_{ijk}$$

We ignored interactions between flat factor, age and (*x*,*y*) (1) because they would be very difficult to interpret, and (2) because of empty cells (in some $f \times age$ combinations) that made the model impossible to solve. *age* was treated as a continuous covariate. Three complete model were fitted, without random effects, then with individual effects (index *j*), then with an interaction between individual and Log(*age*), following Zuur et al. (2009). Tukey's HSD posterior tests were performed on the model to yield the groupings of Fig. A4.1 (Notice that for these variables, groupings apply *within age* only). Specific contrasts were designed to compare particular levels of the flat factor *f* as shown in Table A4.3. For ($D_{max}$, $V_P$, *LD*, *HL*), since only two measurement dates were available, age was included within the flat factor (but appeared in the random effects as a class variable with two levels).

For *destructive variables* ($W$, $W_{abg}$, $W_R$, $W_{str}$, $W_L$, *LA*, $LMA_P$), *age* was included in the flat factor (36 levels) as there were no repeated measures. A fixed-effects linear model was then fitted :

$$\mathrm{Log}(Y_{ij}) = f_i + (\beta x + \gamma y + \delta xy + \zeta x^2 + \eta y^2) + \varepsilon_{ij}$$

Tukey's HSD posterior tests were performed on the model to yield the groupings of Fig. A4.1 (Notice that for these variables, groupings apply over all dates). Specific contrasts were designed to compare particular levels of the flat factor *f* as shown in table 1 (Table A4.4).

For *RGR*, the first method was used as the *RGR* values were computed from repeated measurements. Since the random effects were not significant, we switched back to the second method and treated RGR as the other destructive variables.

## References


Zuur A.F., Ieno E.N., Walker N.J., Saveliev A.A. Smith G.M. 2009. *Mixed effects models and extensions in ecology with R*. Springer, New York

| 'flat' factor levels | F | | | | | | | | | | S | | | | | | | | | |
|---|---|---|---|---|---|---|---|---|---|---|---|---|---|---|---|---|---|---|---|---|
| | CEI | | | | | CYN | | | | | BRI | | | | | PIL | | | | |
| | L | | N | O | | L | | N | O | | L | | N | O | | L | | N | O | |
| **contrast labels** | B | P | P | B | P | B | P | P | B | P | B | P | P | B | P | B | P | P | B | P |
| ecosystem | 1 | 1 | 1 | 1 | 1 | 1 | 1 | 1 | 1 | 1 | -1 | -1 | -1 | -1 | -1 | -1 | -1 | -1 | -1 | -1 |
| forest:shade | 1 | 1 | 0 | -1 | -1 | 1 | 1 | 0 | -1 | -1 | 0 | 0 | 0 | 0 | 0 | 0 | 0 | 0 | 0 | 0 |
| savanna:shade | 0 | 0 | 0 | 0 | 0 | 0 | 0 | 0 | 0 | 0 | 1 | 1 | 0 | -1 | -1 | 1 | 1 | 0 | -1 | -1 |
| forest:fire | 3 | -2 | -2 | 3 | -2 | 3 | -2 | -2 | 3 | -2 | 0 | 0 | 0 | 0 | 0 | 0 | 0 | 0 | 0 | 0 |
| savanna:fire | 0 | 0 | 0 | 0 | 0 | 0 | 0 | 0 | 0 | 0 | 3 | -2 | -2 | 3 | -2 | 3 | -2 | -2 | 3 | -2 |
| light:CEIvsCYN | 1 | 1 | 0 | 0 | 0 | -1 | -1 | 0 | 0 | 0 | 0 | 0 | 0 | 0 | 0 | 0 | 0 | 0 | 0 | 0 |
| light:BRIvsPIL | 0 | 0 | 0 | 0 | 0 | 0 | 0 | 0 | 0 | 0 | 1 | 1 | 0 | 0 | 0 | -1 | -1 | 0 | 0 | 0 |
| shade:CEIvsCYN | 0 | 0 | 0 | 1 | 1 | 0 | 0 | 0 | -1 | -1 | 0 | 0 | 0 | 0 | 0 | 0 | 0 | 0 | 0 | 0 |
| shade:BRIvsPIL | 0 | 0 | 0 | 0 | 0 | 0 | 0 | 0 | 0 | 0 | 0 | 0 | 0 | 1 | 1 | 0 | 0 | 0 | -1 | -1 |
| fire:CEIvsCYN | 1 | 0 | 0 | 1 | 0 | -1 | 0 | 0 | -1 | 0 | 0 | 0 | 0 | 0 | 0 | 0 | 0 | 0 | 0 | 0 |
| fire:BRIvsPIL | 0 | 0 | 0 | 0 | 0 | 0 | 0 | 0 | 0 | 0 | 1 | 0 | 0 | 1 | 0 | -1 | 0 | 0 | -1 | 0 |
| protected:CEIvsCYN | 0 | 1 | 1 | 0 | 1 | 0 | -1 | -1 | 0 | -1 | 0 | 0 | 0 | 0 | 0 | 0 | 0 | 0 | 0 | 0 |
| protected:BRIvsPIL | 0 | 0 | 0 | 0 | 0 | 0 | 0 | 0 | 0 | 0 | 0 | 1 | 1 | 0 | 1 | 0 | -1 | -1 | 0 | -1 |

**Table A4.3**. Contrast coefficients used to test specific factor levels of the 'flat' factor defined for non-destructive variables. Codes as in table A4.1.

| 'flat' factor levels | F | | | | | | | | | | | S | | | | | | | | | | | | | |
|---|---|---|---|---|---|---|---|---|---|---|---|---|---|---|---|---|---|---|---|---|---|---|---|---|---|
| | CEI | | | | | CYN | | | | | | BRI | | | | | | | PIL | | | | | | |
| | 3 | 9 | | 15 | | 3 | 9 | | 15 | | | 3 | 9 | | 15 | | | | 3 | 9 | | 15 | | | |
| | N | L | O | L | | N | L | O | L | O | | N | L | O | L | | O | | N | L | O | L | | O | |
| **contrast labels** | P | P | P | B | P | P | P | P | P | B | P | P | P | P | B | P | B | P | P | P | P | B | P | B | P |
| ecosystem | 14 | 14 | 14 | 14 | 14 | 14 | 14 | 14 | 14 | 14 | 14 | -11 | -11 | -11 | -11 | -11 | -11 | -11 | -11 | -11 | -11 | -11 | -11 | -11 | -11 |
| CEI:date3vs9 | -2 | 1 | 1 | 0 | 0 | 0 | 0 | 0 | 0 | 0 | 0 | 0 | 0 | 0 | 0 | 0 | 0 | 0 | 0 | 0 | 0 | 0 | 0 | 0 | 0 |
| CEI:date9vs15 | 0 | 1 | 1 | -1 | -1 | 0 | 0 | 0 | 0 | 0 | 0 | 0 | 0 | 0 | 0 | 0 | 0 | 0 | 0 | 0 | 0 | 0 | 0 | 0 | 0 |
| CYN:date3vs9 | 0 | 0 | 0 | 0 | 0 | -2 | 1 | 1 | 0 | 0 | 0 | 0 | 0 | 0 | 0 | 0 | 0 | 0 | 0 | 0 | 0 | 0 | 0 | 0 | 0 |
| CYN:date9vs15 | 0 | 0 | 0 | 0 | 0 | 0 | 3 | 3 | -2 | -2 | -2 | 0 | 0 | 0 | 0 | 0 | 0 | 0 | 0 | 0 | 0 | 0 | 0 | 0 | 0 |
| BRI:date3vs9 | 0 | 0 | 0 | 0 | 0 | 0 | 0 | 0 | 0 | 0 | 0 | -2 | 1 | 1 | 0 | 0 | 0 | 0 | 0 | 0 | 0 | 0 | 0 | 0 | 0 |
| BRI:date9vs15 | 0 | 0 | 0 | 0 | 0 | 0 | 0 | 0 | 0 | 0 | 0 | 0 | -2 | -2 | 1 | 1 | 1 | 1 | 0 | 0 | 0 | 0 | 0 | 0 | 0 |
| PIL:date3vs9 | 0 | 0 | 0 | 0 | 0 | 0 | 0 | 0 | 0 | 0 | 0 | 0 | 0 | 0 | 0 | 0 | 0 | 0 | -2 | 1 | 1 | 0 | 0 | 0 | 0 |
| PIL:date9vs15 | 0 | 0 | 0 | 0 | 0 | 0 | 0 | 0 | 0 | 0 | 0 | 0 | 0 | 0 | 0 | 0 | 0 | 0 | 0 | -2 | -2 | 1 | 1 | 1 | 1 |
| forest:shade | 0 | 4 | -5 | 4 | 4 | 0 | 4 | -5 | 4 | -5 | -5 | 0 | 0 | 0 | 0 | 0 | 0 | 0 | 0 | 0 | 0 | 0 | 0 | 0 | 0 |
| savanna:shade | 0 | 0 | 0 | 0 | 0 | 0 | 0 | 0 | 0 | 0 | 0 | 0 | 1 | -1 | 1 | 1 | -1 | -1 | 0 | 1 | -1 | 1 | 1 | -1 | -1 |
| forest:fire | 0 | 0 | 0 | -3 | 2 | 0 | 0 | 0 | 2 | -3 | 2 | 0 | 0 | 0 | 0 | 0 | 0 | 0 | 0 | 0 | 0 | 0 | 0 | 0 | 0 |
| savanna:fire | 0 | 0 | 0 | 0 | 0 | 0 | 0 | 0 | 0 | 0 | 0 | 0 | 0 | 0 | 1 | -1 | 1 | -1 | 0 | 0 | 0 | 1 | -1 | 1 | -1 |
| light:CEIvsCYN | 0 | 2 | 0 | 2 | 2 | 0 | -3 | 0 | -3 | 0 | 0 | 0 | 0 | 0 | 0 | 0 | 0 | 0 | 0 | 0 | 0 | 0 | 0 | 0 | 0 |
| light:BRIvsPIL | 0 | 0 | 0 | 0 | 0 | 0 | 0 | 0 | 0 | 0 | 0 | 0 | 1 | 0 | 1 | 1 | 0 | 0 | 0 | -1 | 0 | -1 | -1 | 0 | 0 |
| shade:CEIvsCYN | 0 | 0 | 3 | 0 | 0 | 0 | 0 | -1 | 0 | -1 | -1 | 0 | 0 | 0 | 0 | 0 | 0 | 0 | 0 | 0 | 0 | 0 | 0 | 0 | 0 |
| shade:BRIvsPIL | 0 | 0 | 0 | 0 | 0 | 0 | 0 | 0 | 0 | 0 | 0 | 0 | 0 | 1 | 0 | 0 | 1 | 1 | 0 | 0 | -1 | 0 | 0 | -1 | -1 |
| fire:CEIvsCYN | 0 | 0 | 0 | 1 | 0 | 0 | 0 | 0 | 0 | -1 | 0 | 0 | 0 | 0 | 0 | 0 | 0 | 0 | 0 | 0 | 0 | 0 | 0 | 0 | 0 |
| fire:BRIvsPIL | 0 | 0 | 0 | 0 | 0 | 0 | 0 | 0 | 0 | 0 | 0 | 0 | 0 | 0 | 1 | 0 | 1 | 0 | 0 | 0 | 0 | -1 | 0 | -1 | 0 |
| protected:CEIvsCYN | 5 | 5 | 5 | 0 | 5 | -4 | -4 | -4 | -4 | 0 | -4 | 0 | 0 | 0 | 0 | 0 | 0 | 0 | 0 | 0 | 0 | 0 | 0 | 0 | 0 |
| protected:BRIvsPIL | 0 | 0 | 0 | 0 | 0 | 0 | 0 | 0 | 0 | 0 | 0 | 1 | 1 | 1 | 0 | 1 | 0 | 1 | -1 | -1 | -1 | 0 | -1 | 0 | -1 |

**Table A4.4**. Contrast coefficients used to test specific factor levels of the 'flat' factor defined for destructive variables. Codes as in table A4.1.

**Figure A4.1**. Estimates of fixed effects for the variables $L_P$, $n_L$, $H_{max}$, $n_A$, $D_{max}$, $V_P$, $W$, $W_{abg}$, $W_R$, $W_{str}$, $W_L$, $LA$, $LD$, $HL$, $LMA_P$, $rs$, $ls$, $RGR$. Error bars represent one standard error (for models with random effects, only the residual error is considered). Bars with different letters are significantly different at the 5 % level; for variables with repeated measures ($L_P$, $n_L$, $H_{max}$, $n_A$, $HL$), the letters do not include the date effect, which was treated as a covariate. Numbers on bars are the number of values used to compute every effect.

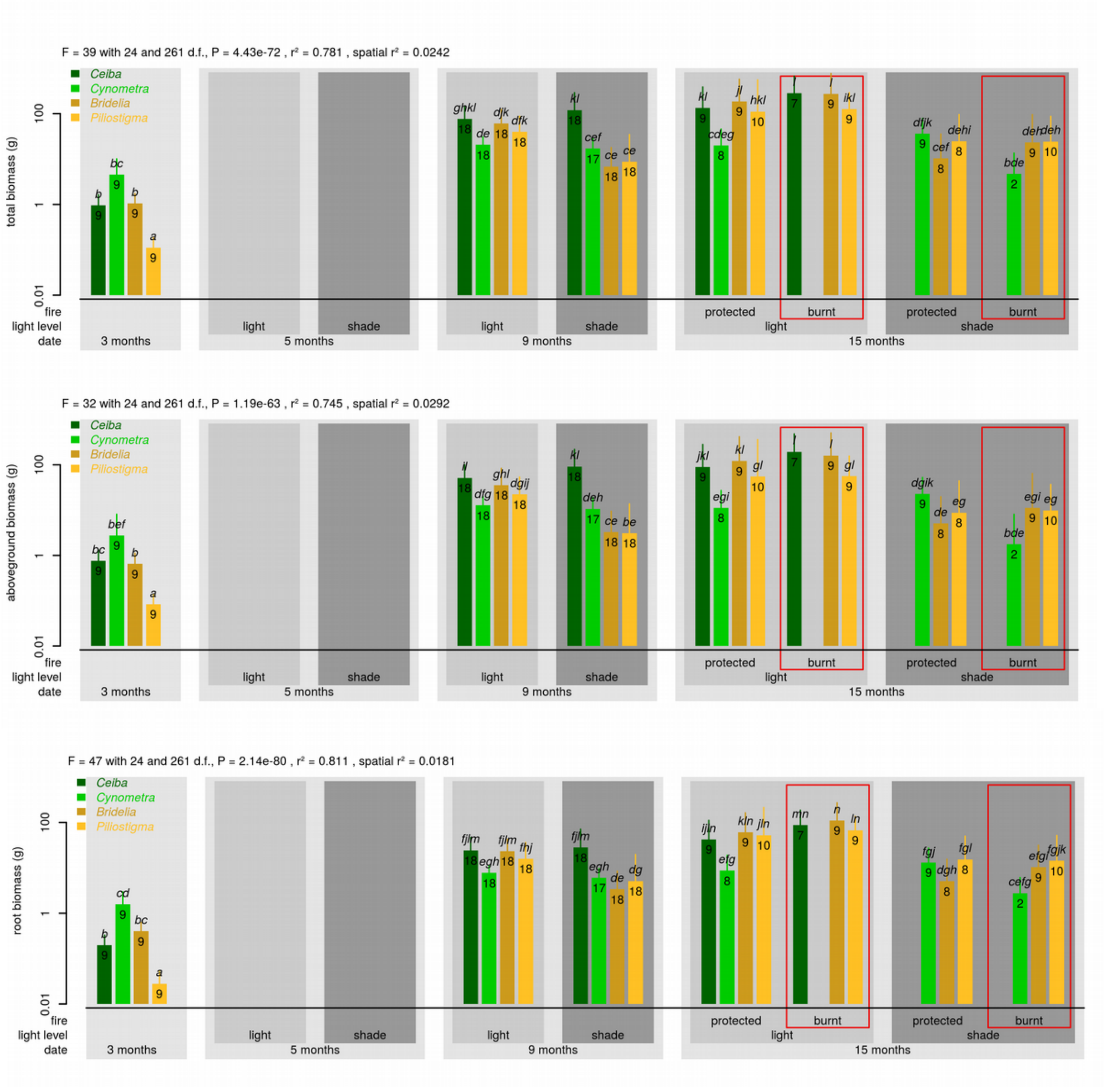

**Figure A4.1,** continued

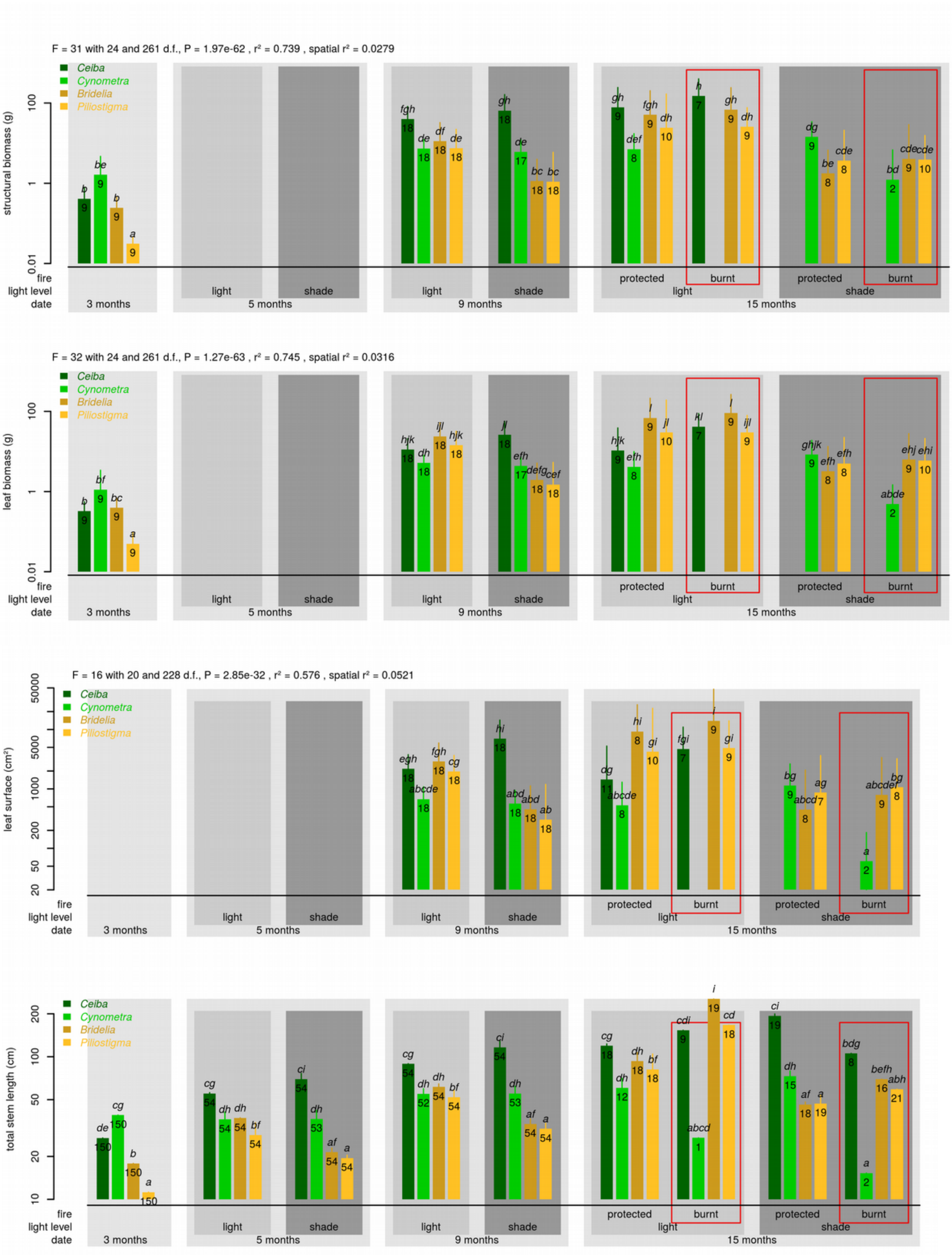

**Figure A4.1,** continued

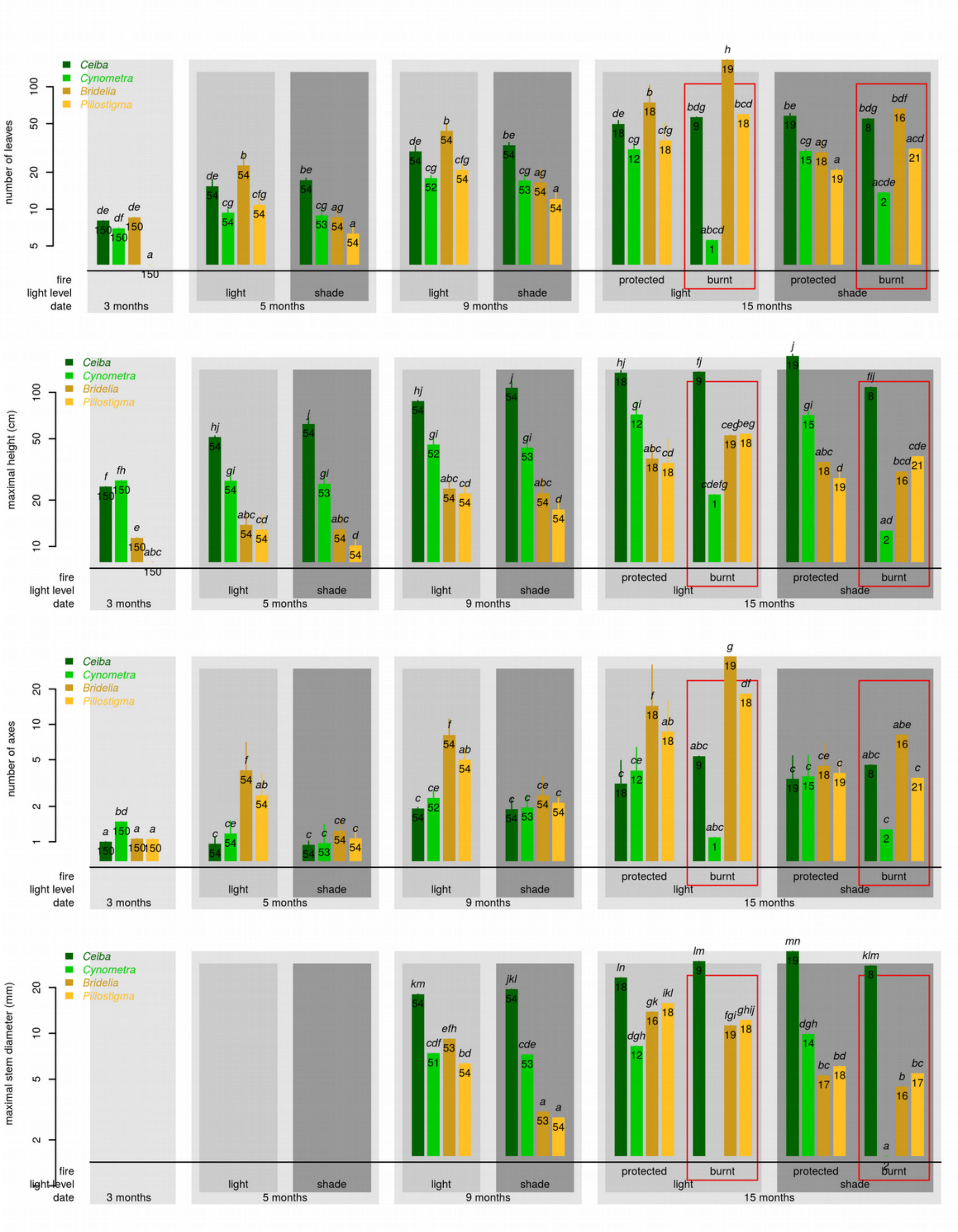

**Figure A4.1,** continued

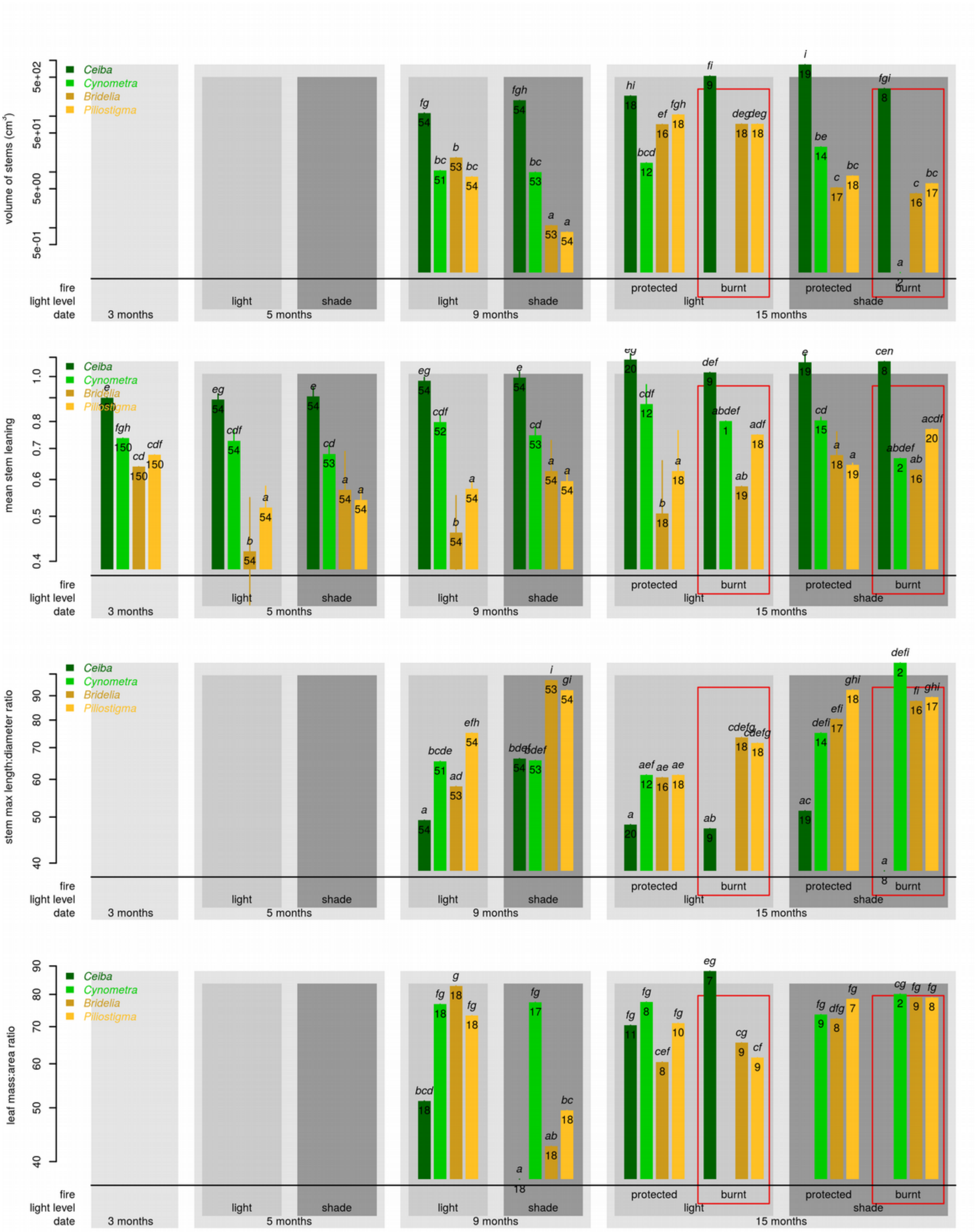

**Figure A4.1**, continued

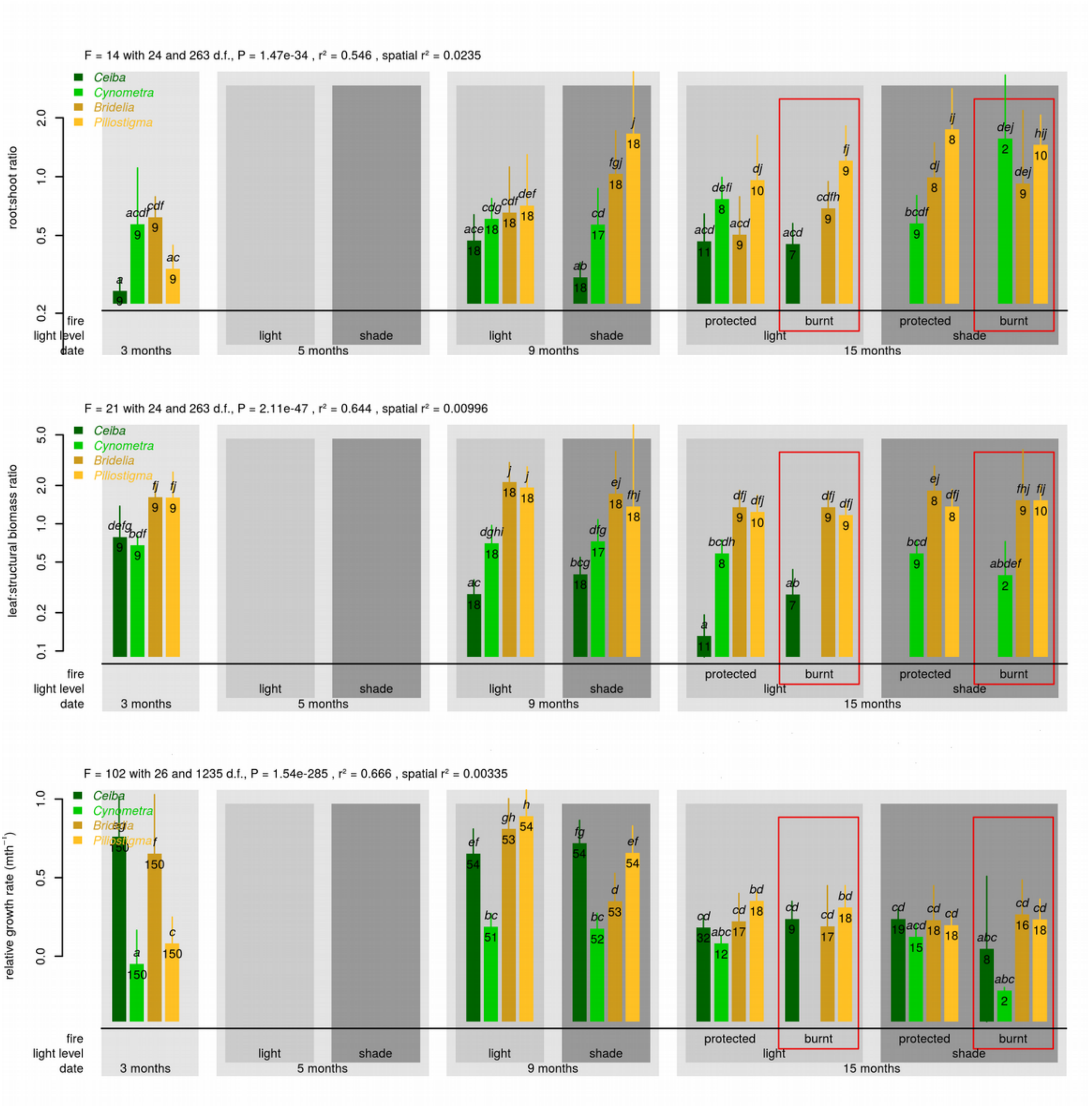

# Appendix 5. Analysis of survival to fire

**Table A5.1.** Analysis of survival. Best-fit generalized linear model with binomial error and logit link function (R glm function) of survival at date 15 after stepwise deletion of non-signficant terms. All remaining effects are significant at the 0.05 level. The initial model included the root:total biomass ratio and all interactions up to 3rd order. From the evidence of Fig. A5.1, the shade treatement was not included in the initial model.

| | d.f. | Deviance | Residual d.f. | Residual deviance | Pr(>$\chi^2$) |
|---|---|---|---|---|---|
| error | | | 281 | 304.49 | |
| ecosystem | 1 | 69.99 | 280 | 234.50 | <0.001 |
| fire | 1 | 56.61 | 279 | 177.89 | <0.001 |
| Log($\hat{W}$) | *3* | 39.46 | 278 | 138.43 | <0.001 |
| ecosystem:fire | 1 | 7.94 | 277 | 130.49 | 0.001 |

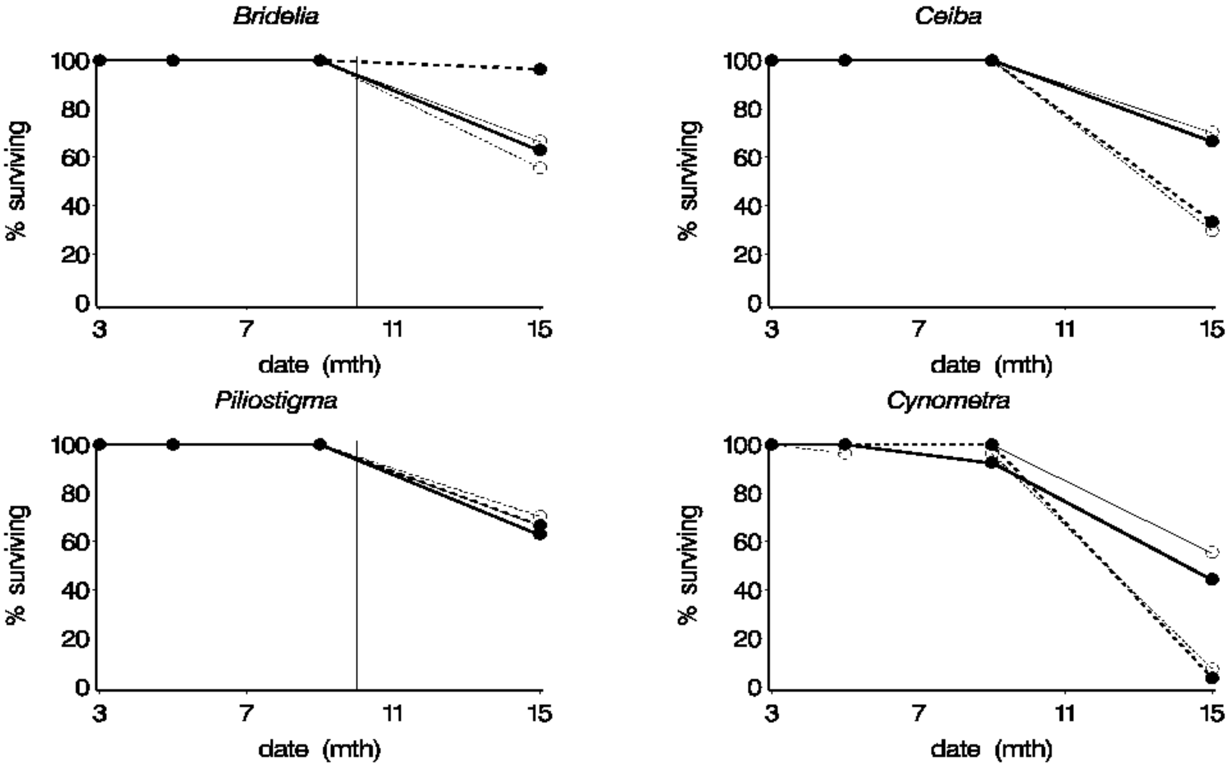


**Figure A5.1**. Survival of seedlings during the experiment. Seedlings dying between dates 3 (age of the seedlings when they were transplanted in the field) and 5 were replaced (except one *Cynometra*), assuming their death was due to transplantation. Fire occured at month 10 (vertical line). Cohorts all initially comprised 27 seedlings. Treatments: thick lines, full light; thin lines, shade; solid lines, unburnt; dotted lines, burnt.
